# Transcription factor MITF regulates masseter muscle growth and development

**DOI:** 10.14814/phy2.70677

**Published:** 2025-11-24

**Authors:** Megumi Nariyama, Yoshiki Ohnuki, Kenji Suita, Misao Ishikawa, Ren Matsubara, Ichiro Matsuo, Takao Mitsubayashi, Yasumasa Mototani, Aiko Ito, Mariko Abe, Yoshio Hayakawa, Takako Nomura, Satoshi Wada, Yoshinobu Asada, Satoshi Okumura

**Affiliations:** ^1^ Department of Pediatric Dentistry Tsurumi University School of Dental Medicine Yokohama Japan; ^2^ Department of Physiology Tsurumi University School of Dental Medicine Yokohama Japan; ^3^ Department of Anatomy Tsurumi University School of Dental Medicine Yokohama Japan; ^4^ Department of Oral and Maxillofacial Surgery Ibaraki Medical Center Tokyo Medical University Ibaraki Japan; ^5^ Department of Periodontology Tsurumi University School of Dental Medicine Yokohama Japan; ^6^ Department of Orthodontics Tsurumi University School of Dental Medicine Yokohama Japan; ^7^ Department of Dental Anesthesiology Tsurumi University School of Dental Medicine Yokohama Japan; ^8^ Department of General Dentistry and Clinical Education Tsurumi University School of Dental Medicine Yokohama Japan; ^9^ Department of Oral and Maxillofacial Facial Surgery School of Medicine, Kanazawa Medical University Uchinada Ishikawa Japan

**Keywords:** cell signaling, microRNA, skeletal muscle

## Abstract

The microphthalmic mouse with a mutation at the locus of the microphthalmia‐associated transcriptional factor (MITF) gene exhibits masticatory dysfunction due to impaired masseter muscle development and needs to be fed with powdered diet. However, the effects of MITF mutation on masseter muscle remain poorly understood. Here, we show that masseter muscle mass is markedly decreased in MITF‐mutant mice (*mi*/*mi*), in contrast to the corresponding tibialis anterior and soleus muscles. The area of fibrosis and degree of myocyte apoptosis were strikingly increased in the masseter muscle of *mi*/*mi*. The expression of muscle‐specific microRNAs (miR‐1, miR‐206, and miR‐133a), which are necessary for proper skeletal muscle development and function, was strongly suppressed in the masseter muscle of *mi/mi* during development. In addition, the regulation of reactive oxygen species production, calcium homeostasis via sarcoendoplasmic reticulum calcium transport and autophagic activity, which are important for maintaining skeletal muscle mass and function, were all altered in the masseter muscle of *mi*/*mi*. These results indicate that MITF is required for masseter muscle growth and development.

## 1 INTRODUCTION

Microphthalmia‐associated transcriptional factor (MITF) is a member of the basic helix‐loop‐helix leucine zipper (bHLH‐Zip) family of DNA‐binding proteins (Hodgkinson et al., 1993). Nine isoforms have been identified in mice, MITF‐A, ‐B, ‐C, ‐D, ‐E, ‐H, ‐J, ‐M, and ‐mc, in which the first exon is isoform‐specific, while exons 2–9 are identical. MITF isoforms are expressed in a tissue‐specific manner, and their transcriptional activities differ (Amae et al., 1998; Bharti et al., 2008; Funaba et al., 2005; Murakami et al., 2003; Takemoto et al., 2002).

The major isoform of skeletal muscle is MITF‐A, while the major cardiac isoform is MITF‐H. MITF‐A is also the second most abundant isoform in melanocytes and plays a role in pigmentation of the hair and eye; mice lacking MITF‐A exhibit albinism (Flesher et al., 2020; Katayama et al., 2010).

In a C2C12 myotube differentiation model, C2C12 myoblasts fuse to form multinucleated myotube cells upon serum starvation (Yaffe & Saxel, 1977). Expression of MITF‐A was increased after differentiation, suggesting that MITF‐A might be a positive regulator of myogenesis (Ooishi et al., 2012).

Studies using MITF‐mutant mice (*mi*/*mi*) have shown that cardiac muscle weight is decreased at baseline, and hypertrophy induced by β‐adrenergic receptor (β‐AR) stimulation with isoproterenol or angiotensin‐II is also decreased (Liu et al., 2014; Tshori et al., 2006). Cardiac function in terms of echocardiography is significantly decreased in *mi/mi*, compared to control wild‐type (WT) mice (Tshori et al., 2006) and MITF expression in the heart is decreased in canines with tachycardia‐induced heart failure (Gao et al., 2006). Nevertheless, the role of MITF in skeletal muscle in vivo remains poorly understood.

microRNAs (miRNAs) are a class of highly conserved small non‐coding RNAs that negatively regulate gene expression post‐transcriptionally (Nariyama et al., 2015). In particular, muscle‐specific miR‐1 and miR‐206 promote myogenesis, while miR‐133a promotes myoblast proliferation in C2C12 cells (Townley‐Tilson et al., 2010). Further, we previously reported that the expression of miR‐1 in masseter muscle (MA) at 12 weeks was 12.3‐fold higher than that at 1 week, whereas in the gastrocnemius, it was only 7.4‐fold higher, suggesting that miR‐1 might play a more prominent role for muscle growth and development in MA than in gastrocnemius muscle (Nariyama et al., 2012).

We have long been interested in the physiology of MA, because masticatory dysfunction impacts food selection and nutrition, which play pivotal roles in ensuring good general health and quality of life (Lahoud et al., 2023). We previously examined the role of acetylcholine receptor clustering in MA (Mori et al., 2013; Nariyama et al., 2015) and the response to external stresses such as mechanical overload (Nariyama et al., 2012; Umeki et al., 2013), and chronic catecholamine stress (Ito et al., 2019; Ohnuki et al., 2013, 2014; Umeki et al., 2015). Here, we focused on the role of MITF in the growth and development of MA, because the effects of MITF mutation on MA remain poorly understood, even though mice with a defective MITF gene exhibit masticatory dysfunction due to impaired MA development, and need to be fed a powdered diet. In this study, therefore, we used MITF‐mutant mice to examine the role of MITF in the growth and development of MA. In addition, we examined whether the MA phenotypes in these mice might be recapitulated in other skeletal muscles such as tibialis anterior (TA) or soleus muscle (SOL).

## 2 METHODS

### 2.1 Mice

**FIGURE 1:**
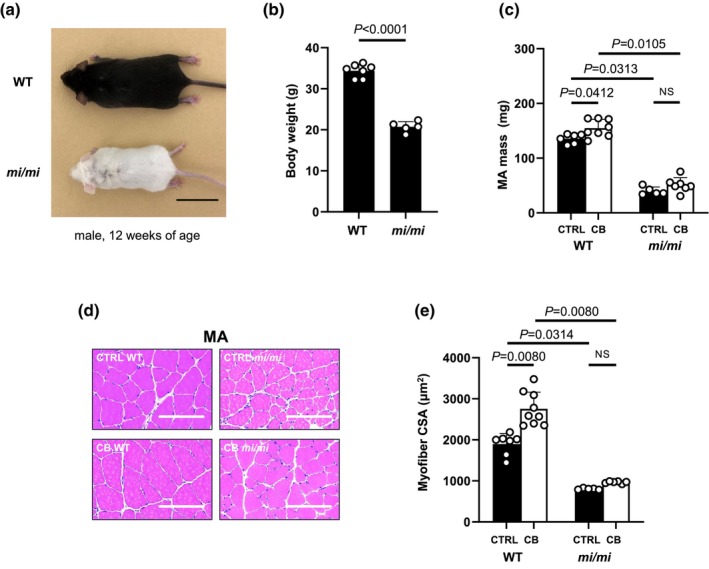
Effects of MITF mutation on body weight, MA weight and cross‐sectional area. (a) Pictures of male wild‐type (WT; upper) and MITF mutant mice (*mi/mi*; lower) at 12 weeks of age. (b) Body weight was significantly smaller in *mi/mi* than in WT (WT [*n* = 7 each], *mi/mi* [*n* = 5 each], ***p < 0.001 by Student's *t*‐test). (c) MA mass was significantly increased by CB treatment in WT (control WT [*n* = 6] vs. CB WT [*n* = 8]: *p* = 0.0412; ANOVA/Tukey–Kramer) but the increase was suppressed in *mi/mi* (control *mi/mi* [*n* = 5] vs. CB *mi/mi* [*n* = 7], *p* = NS; *not significant*). Age‐matched control mice (CTRL) received an identical volume of saline only. (d) Representative images of hematoxylin–eosin‐stained sections of masseter muscle (MA) in the control (CTRL) WT (*upper left*), CTRL *mi/mi* (*upper right*), CB‐treated (CB) WT (*lower left*) and CB *mi/mi* (*lower right*). (e) The cross sectional area (CSA) was significantly increased by CB treatment in WT (CTRL WT [*n* = 7] vs. CTRL *mi/mi* [*n* = 9], *p* = 0.0080; ANOVA/Tukey–Kramer) but the increase was suppressed in *mi/mi* (CTRL‐*mi/mi* [*n* = 5] vs. CB‐*mi/mi* [*n* = 6], *p* = NS; ANOVA/Tukey–Kramer).

A breeding pair of mice heterozygous for the MITF mutation (*mi/*+ male and *mi/*+ female) (strain name, B6C3Fe a/a‐Mitfmi/J) was purchased from the Jackson Laboratory (Bar Harbor, ME, USA) (Lin et al., 2004; Weilbaecher et al., 2001), and homozygous mutant (*mi/mi*) and WT mice were then developed by mating heterozygous MITF mutants. Note the white coat color of the *mi*/*mi* (Figure 1a; *lower*); this is because the homozygous mutations at the MITF locus result in pigmentation defects, as described previously, so that the mice used for comparison in this study have different coat colors, even though they have the same background (Figure 1a) (Tachibana, 2000). The pups were breast‐fed and removed from their dams at about 3 weeks after birth. After being weaned, the mice were fed a commercial powdered standard diet (CE‐2: 334 kcal/100 g; CLEA, Tokyo, Japan).

Clenbuterol (CB; #C5423, Sigma, St. Louis, MO, USA) was administered i.p. once daily for 3 weeks, starting at the age of 12 weeks, and control mice received an identical volume of saline only (Ohnuki et al., 2014; Ohnuki et al., 2016).

The *mi/mi* and WT were killed by cervical dislocation under isoflurane (1.0%–1.5% v/v) anesthesia delivered via a mask at room temperature at 15 weeks of age, and the left and right MAs were dissected out. The excised MA, TA and SOL muscles were weighed, then immediately frozen in liquid nitrogen with Tissue‐Tek OCT compound (Sakura Finetek, Torrance, CA, USA), and stored at −80°C until sectioning.

### 2.2 Myocyte cross‐sectional area

To avoid the influence of compartmental specialization within the MA, cross sections (10 μm) were cut from the middle portion of the left masseter excised from mice at 15 weeks of age with a cryostat (CM1900, Leica Microsystems, Nussloch, Germany) at −20°C (Ito et al., 2019; Ohnuki et al., 2014; Urushiyama et al., 2004; Zhao et al., 2024).

The sections were air‐dried, fixed with 4% paraformaldehyde (v/v) in 0.1 M phosphate‐buffered saline (pH 7.5), stained with hematoxylin and eosin, and observed under a light microscope (BX61, Olympus Co., Tokyo, Japan). Micrographs were taken with a digital camera (DP‐72, Olympus Co.) connected to a personal computer. The cross‐sectional size of muscle fibers was evaluated by measuring the cross‐sectional area (CSA). The CSA of 90–200 myocytes was measured with image analysis software (ImageJ 1.45) and averaged to obtain the mean value in each mouse (Ito et al., 2019, 2023).

### 2.3 Histological analysis

We employed Masson‐trichrome staining using the Accustain Trichrome Stain kit (#HT15‐1KT; Sigma‐Aldrich, St. Louis, MO, USA) in accordance with the manufacturer's protocol (Suita et al., 2020). In Masson‐trichrome staining, fibers stained in aniline blue are collagen fibers, while those stained in red are muscle fibers. Sections of the MA tissues excised from mice at 15 weeks of age were outlined manually to define regions of interest (ROIs) (control WT [*n* = 6], control *mi*/*mi* [*n* = 5], CB‐treated WT [*n* = 9] and CB‐treated *mi*/*mi* [*n* = 6]). We measured the percentage fibrosis within 3–5 ROIs for each section, using ImageJ 1.48v software (National Institute of Health, Bethesda, MD, USA https://imagej.nih.gov/ij/download.html) (Ito et al., 2019).

DNA fragmentation was determined by terminal deoxyribonucleotidyl transferase (TdT)‐mediated biotin‐16‐deoxyuridine (TUNEL) staining using an Apoptosis in situ Detection kit (#293‐71501; FUJIFILM Wako Pure Chemical Corporation, Osaka, Japan). The total number of TUNEL‐positive nuclei was counted manually in six sections in the four groups (control WT [*n* = 6], control *mi*/*mi* [*n* = 6], CB‐treated WT [*n* = 6], and CB‐treated *mi/mi* [*n* = 6]).

The number of peripheral and centralized nuclei was counted in five random non‐overlapping microscopic fields in sections stained with hematoxylin and eosin (Yu et al., 2014). More than 400 myofibers were counted for each sample.

### 2.4 Western blotting

MA tissue excised from the mice at 15 weeks of age was homogenized in a polytron (Kinematica AG, Lucerne, Switzerland) in ice‐cold RIPA buffer (#89900; Thermo Fisher Scientific, Waltham, MA, USA: 25 mM Tris–HCl (pH 7.6), 150 mM NaCl, 1% sodium deoxycholate, 0.1% SDS) with the addition of Halt™ Protease Inhibitor Cocktail, EDTA‐free (#87785; Thermo Fisher Scientific) and the homogenate was centrifuged at 13,000×*g* for 10 min at 4°C. The supernatant was collected and the protein concentration was measured using a DC protein assay kit (#5000111; Bio‐Rad, Hercules, CA, USA). Equal amounts of protein (5 μg) were subjected to 12.5% SDS‐polyacrylamide gel electrophoresis and blotted on PVDF membrane (#IPVH00010; Millipore, Burlington, MA, USA).

Western blotting was conducted with commercially available antibodies (Okumura, Kawabe, et al., 2003; Okumura et al., 2007, 2014; Okumura, Takagi, et al., 2003). Primary antibodies against caspase‐3 (1:500, #9662), caspase‐9 (1:1000, #9504), Akt (1:1000, #9272), phospho‐Akt (1:1000, Ser‐473, #9721), calcium/calmodulin‐dependent protein kinase II (CaMKII) (1:1000, #3362), Bcl‐2 associated protein (Bax) (1:1000, #2772), microtube‐associated protein light chain 3 (LC3) (1:1000, #12741), B cell lymphoma 2 (Bcl‐2) (1:1000, #3498), phospho‐mechanistic target of rapamycin (mTOR) (1:1000, Ser‐2448, #5536), mTOR (1:1000, #2972), p44/42 mitogen‐activated protein kinase (MAPK) (also known as ERK) (1:1000, #4695), phospho‐ERK (1:2000, Thr‐202/Tyr‐204, #4370), α‐smooth muscle actin (α‐SMA) (1:1000, #19245) were purchased from Cell Signaling Technology (Boston, MA, USA), primary antibody against glyceraldehyde‐3‐phosphate dehydrogenase (GAPDH) (1:200, sc‐25778) was purchased from Santa Cruz Biotechnology (Santa Cruz, CA, USA) and primary antibodies against phospholamban (PLB) (1:5000, #A010‐14), phospho‐PLB (1:5000, Thr‐17, #A010‐13; 1:5000, Ser‐16, A010‐12) were purchased from Badrilla (Leeds, UK). Primary antibodies against nicotinamide adenine dinucleotide phosphate oxidase (Nox) 4 (1:1000, #ab133303) and Nox2 (1:1000, #ab80508) were purchased from Abcam (Cambridge, UK) and primary antibody against oxidized CaMKII (ox‐CaMKII) (Met‐281/282) (1:1000, #07‐1387) was purchased from Millipore (Billerica, MA, USA). The primary antibodies against p62 (1:1000, #PM045) and phospho‐p62 (1:500, Ser‐351) (#PM074MS) were purchased from MBL (Nagoya, Japan). Horseradish peroxide‐conjugated anti‐rabbit (1:5000, #NA934) or anti‐mouse IgG (1:5000, #NA931) antibodies purchased from GB Healthcare (Amersham, UK) were used as secondary antibodies. The primary and secondary antibodies were diluted in Tris‐buffered saline (pH 7.6) with 0.1% Tween 20 and 5% bovine serum albumin. Protein oxidation was measured using the OxiSelect™Protein Carbonyl Immunoblot Kit (#STA‐308, Cell Biolabs, Inc. San Diego, CA, USA) according to the manufacturer's instructions (Hwee et al., 2014; Tanase et al., 2016). The blots were visualized with enhanced chemiluminescence solution (#RPN2232, ECL Prime Western Blotting Detection Reagent, GE Healthcare, Piscataway, NJ, USA) and scanned with a densitometer (LAS‐1000, Fuji Photo Film, Tokyo, Japan). The amount of expression in the Control was taken as 100% in each determination in accordance with previous studies (Okumura et al., 2007, 2014).

### 2.5 Immunostaining

Oxidative DNA damage in the myocardium was evaluated by immunostaining for 8‐hydroxy‐2′‐deoxyguanosine (8‐OHdG) using the Vector M.O.M. Immunodetection system (#PK‐2200, Vector Laboratories, Inc. Burlingame, CA, USA) (Suita et al., 2020; Yagisawa et al., 2020). Cross sections (WT: *n* = 6, *mi/mi*: *n* = 6) were cut from the middle portion of the left masseter excised from mice at 15 weeks of age with a cryostat (CM1900, Leica Microsystems, Nussloch, Germany) (Ito et al., 2019; Ohnuki et al., 2014; Urushiyama et al., 2004; Zhao et al., 2024), air‐dried and fixed with 4% paraformaldehyde (v/v) in tris‐buffered saline (TBS)‐T for 5 min at room temperature. Antigen retrieval was achieved with 0.1% citrate plus 1% Triton X‐100 for 30 min at room temperature; then the sections were washed with TBS‐T, incubated with 0.3% horse serum in TBS‐T for 1 h at room temperature, and blocked with M.O.M. blocking reagents (Vector Laboratories, Burlingame, CA, USA) overnight at 4°C. For the positive control, sections were incubated with 0.3% H_2_O_2_ in TBS‐T before the anti‐8‐OHdG antibody treatment. The sections were incubated with anti‐8‐OHdG antibody (8.3 μg/mL in M.O.M. Dilute; clone N45.1 monoclonal antibody; #MOG‐020P; Japan Institute for the Control of Aging, Shizuoka, Japan) overnight at 4°C in a humidified chamber, and then incubated with 0.3% H_2_O_2_ in 0.3% horse serum for 1 h at room temperature to inactivate endogenous peroxidase, rinsed with TBS‐T, incubated with anti‐horse IgG in M.O.M. Dilute, and processed with an ABC kit (PK‐4000; Vector Laboratories, Inc. Burlingame, CA, USA). We calculated the ratio of 8‐OHdG nuclei with oxidative DNA damage (stained dark brown) per total cell number.

### 2.6 miRNA expression analysis by real‐time PCR

Total miRNA was extracted from the MA excised from mice at 15 weeks of age using a High Pure miRNA Isolation Kit (#05 080 576 001, Roche Diagnostics GmbH, Roche Applied Science, Mannheim, Germany) at 1, 4, 8, and 12 weeks of age. Reverse transcription was performed using the TaqMan MicroRNA Reverse Transcription Kit (#4366596, Applied Biosystems, Foster City, CA, USA). All cDNA samples were stored at −20°C until required. To quantify the miRNA expression, real‐time polymerase chain reaction (PCR) was performed using the Thermal Cycler Dice® Real Time System (Takara Bio Inc., Shiga, Japan) and the TaqMan microRNA Assay primers for miR‐1, miR‐24, miR‐133a and miR‐206 (Applied Biosystems). Amplification of snoRNA‐202 served as an endogenous control to normalize the expression of the target miRNAs. The thermocycling conditions were 95°C for 10 min, followed by 40 cycles of 95°C for 15 s and 60°C for 60 s.

The sequences of the primers were: mmu‐miR 24‐UGGCUCAGUUCAGCAGGAACAG; snoRNA202‐GCTGTACTGACTTGATGAAAGTACTTTTGAACCCTTTTCCATCTGATG; mmu‐miR‐1 forward GGAAACATACTTCTTTAT and reverse ACTTCTTTACATTCCATA; mmu‐miR‐133a forward TGCTGAAGCTGGTAAAATGGA and reverse GCTGGTTGAAGGGGACCaA; mmu‐mir‐206 forward AGGCCACATGCTTCTTTATA and reverse TTCCTTACATTCCATAGTGC.

### 2.7 Statistical analysis

**FIGURE 2:**
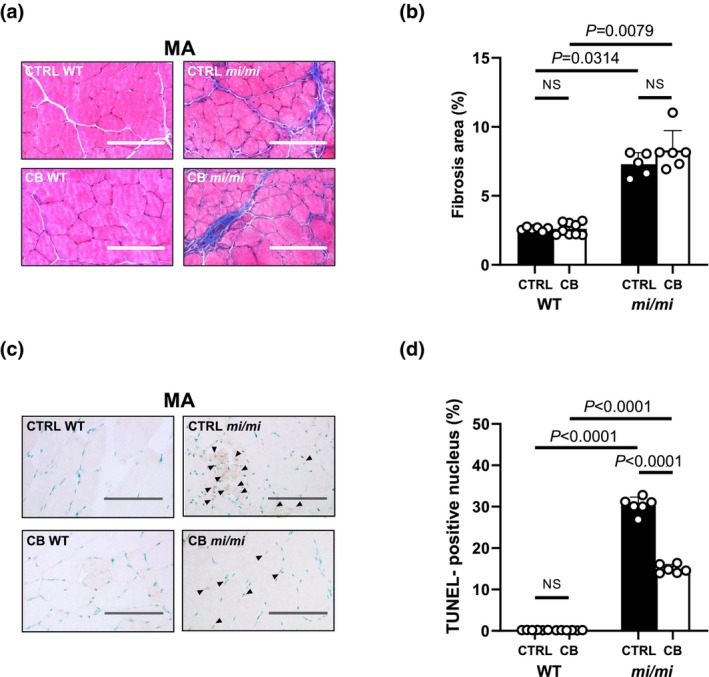
Effects of MITF mutation on fibrosis and myocyte apoptosis in MA. (a) Representative images of Masson‐trichrome‐stained sections of masseter muscle (MA) in the control (CTRL) WT (*upper left*), CTRL *mi/mi* (*upper right*), CB WT (*lower left*) and CB *mi/mi* (*lower right*). (b) CB treatment did not alter the area of fibrosis in either WT or *mi/mi* (*P* = NS each; Kruskal–Wallis/Steel‐Dwass). However, the area of fibrosis was approximately 3‐fold greater in both the control and CB‐treated group in *mi/mi*, compared to WT (Control: CTRL WT [*n* = 6] vs. CTRL *mi/mi* [*n* = 5]; *p* = 0.00314; Kruskal–Wallis/Steel‐Dwass; CB WT [*n* = 9] vs. CB *mi/mi* [*n* = 6]; *p* = 0.0079; Kruskal–Wallis/Steel‐Dwass). (c) Representative images of TUNEL‐stained sections of MA in the control (CTRL) WT (*upper left*), CTRL *mi/mi* (*upper right*), CB WT (*lower left*) and CB *mi/mi* (*lower right*). (d) The number of TUNEL‐positive myocytes was similar in MA of WT with/without CB treatment (CTRL WT [*n* = 6] vs. CB WT [*n* = 6]; *p* = NS; ANOVA/Tukey–Kramer). However, the number of TUNEL positive myocytes was significantly increased in MA of *mi/mi* with/without CB treatment (CTRL WT [*n* = 6] vs. CTRL *mi/mi* [*n* = 6]; *p* < 0.0001; ANOVA/Tukey–Kramer), but its magnitude was significantly decreased in CB‐treated *mi/mi* mice (*p* < 0.0001; ANOVA/Tukey–Kramer)

**FIGURE 3:**
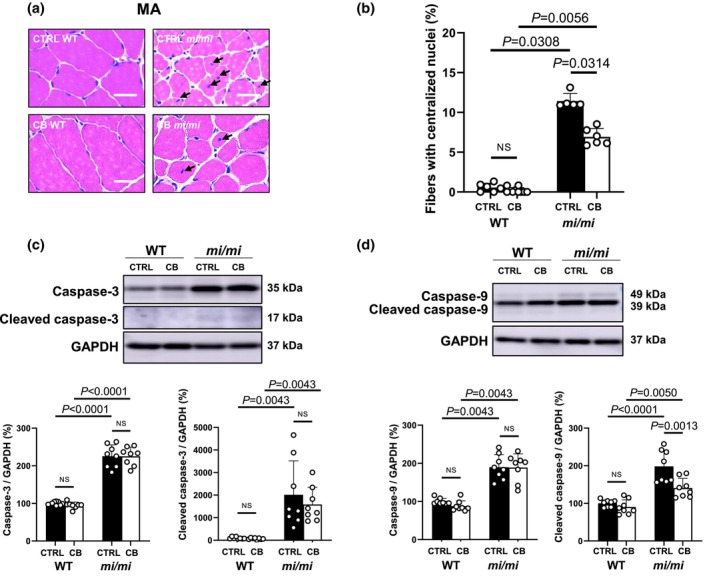
Effects of MITF mutation on the number of myofibers with centralized nuclei and the expression of caspase 3/9 in MA. (a) Representative images of hematoxylin–eosin‐stained sections of MA in the control (CTRL) WT (*upper left*), CTRL *mi/mi* (*upper right*), CB WT (*lower left*) and CB *mi/mi* (*lower right*). (b) The percentage of fibers with centralized nuclei was similar in MA of WT with/without CB treatment (CTRL WT TUNEL‐positive myocytes similar in MA of WT with/without CB treatment (CTRL WT [*n* = 6] vs. CB WT [*n* = 9]; *p* = NS; Kruskal–Wallis/Steel‐Dwass). However, the percentage of fibers with centralized nuclei was significantly increased in MA of *mi/mi* with/without CB treatment (CTRL WT [*n* = 6] vs. CTRL *mi/mi* [*n* = 5]; *p* = 0.0308; Kruskal–Wallis/Steel‐Dwass; CB WT [*n* = 9] vs. CB *mi/mi* [*n* = 6]; *p* = 0.0056; Kruskal–Wallis/Steel‐Dwass) and the magnitude of the increase was much greater in CTRL *mi/mi*, compared to CB *mi/mi* (CTRL *mi/mi* [*n* = 5] vs. CB *mi/mi* [*n* = 6], *p* = 0.0314; Krsukal–Wallis/Steel‐Dwass). (c) Expression levels of full‐length and cleaved caspase‐3 were similar with/without CB treatment in MA of WT (full‐length: *p* = NS; ANOVA/Tukey–Kramer; cleaved: *p* = NS; Kruskal–Wallis/Steel‐Dwass). However, the expression levels of full‐length and cleaved caspase‐3 in *mi/mi* mice were significantly increased in both the control (full‐length: *p* < 0.0001; ANOVA/Tukey–Kramer; cleaved: *p* = 0.0043; Kruskal–Wallis/Steel‐Dwass) and the CB‐treated group (full‐length: *p* < 0.0001; ANOVA/Tukey–Kramer; cleaved: *p* = 0.0043; Kruskal–Wallis/Steel‐Dwass). (d) Expression levels of full length and cleaved caspase‐9 were similar with/without CB treatment in WT (full length: *p* = NS; Kruskal–Wallis/Steel‐Dwass; cleaved: *p* = NS; ANOVA/Tukey–Kramer). However, the expressions of full‐length and cleaved caspase‐9 in *mi/mi* mice were significantly increased in both the control (full‐length: *p* = 0.0043; Kruskal–Wallis/Steel‐Dwass; cleaved: *p* < 0.0001; ANOVA/Tukey–Kramer) and the CB‐treated group (full‐length: *p* = 0.0043; Kruskal–Wallis/Steel‐Dwass; cleaved: *p* = 0.0050;ANOVA/Tukey–Kramer).

**FIGURE 4:**
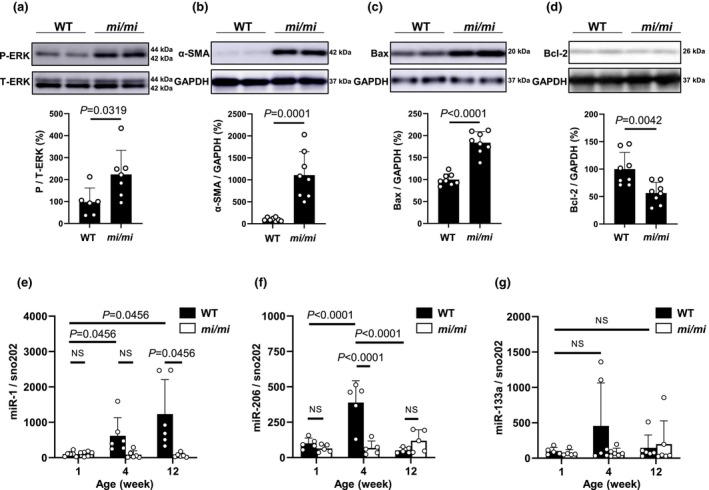
Effects of MITF mutation on expression of phospho‐ERK, α‐SMA, Bax and Bcl‐2, miR‐1, miR‐206 and miR‐133 in MA. (a) Expression of phospho‐ERK (Thr202/Tyr204) was significantly increased in MA of *mi/mi* [*n* = 7], compared to WT [*n* = 6] (*p* = 0.0319; Student's *t*‐test). Images of full‐size immunoblots are shown in Data S2. (b) Expression of α‐SMA was significantly increased in MA of *mi/mi* [*n* = 8], compared to WT [*n* = 8] (*p* = 0.0001; Student's *t*‐test). Images of full‐size immunoblots are shown in Data S3. (c) Expression of Bax was significantly increased in MA of *mi/mi* [*n* = 8], compared to WT (*n* = 8) (*p* < 0.0001; Student's *t*‐test). Images of full‐size immunoblots are shown in Data S2. (d) Expression of Bcl‐2 was significantly decreased in MA of *mi/mi* [*n* = 8], compared to WT [*n* = 8] (*p* = 0.0042; Student's *t*‐test). Images of full‐size immunoblots are shown in Data S2. (e) Expression of miR‐1 in MA was similar in both WT [*n* = 6] and *mi/mi* [*n* = 6] at 1 week after birth. It was significantly increased at 4 weeks (*p* = 0.0456; Kruskal–Wallis/Steel‐Dwass)) and further increased at 12 weeks (*p* = 0.0456; Kruskal–Wallis/Steel‐Dwass) after birth in WT [*n* = 6], but these increases were suppressed in *mi/mi* [*n* = 6]. (f) Expression of miR‐206 in MA was similar in both WT [*n* = 5] and *mi/mi* [*n* = 5] at 1 week after birth (*p* = NS; ANOVA/Tukey–Kramer). It was significantly increased at 4 weeks in WT [*n* = 5] (*p* < 0.0001; ANOVA/Tukey–Kramer), but the increase was suppressed in *mi/mi* [*n* = 5]. (g) Expression of miR‐133 in MA was depressed in both WT and *mi/mi* during development (*p* = NS; Kruskal–Wallis/Steel‐Dwass).

**FIGURE 5:**
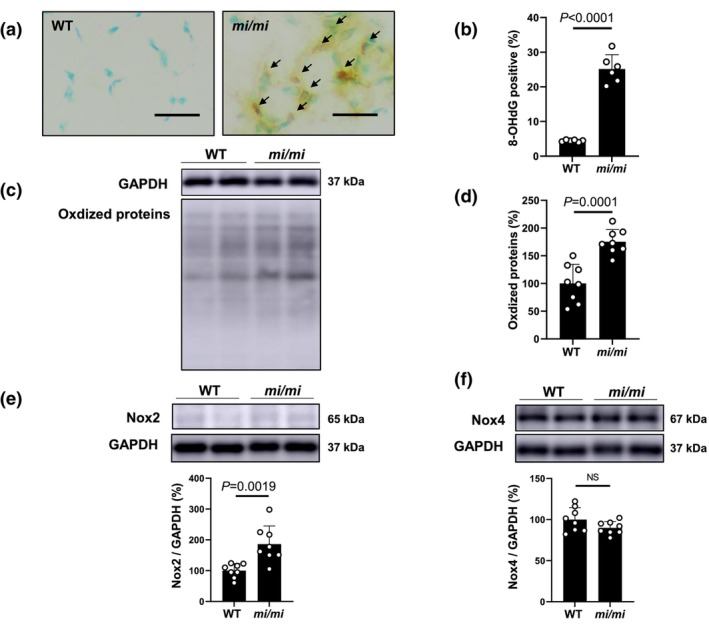
Effects of MITF mutation on oxidative stress in MA. (a) Representative immunohistochemical images of oxidative DNA damage (8‐OHdG) in MA in WT (*left*) and *mi/mi* (*right*). (b) 8‐OHdG‐positive nuclei were significantly increased in *mi/mi* (*n* = 6), compared to WT (*n* = 6) (*p* < 0.0001; Student's *t*‐test). (c) Representative SDS‐PAGE of oxidized proteins in MA homogenate prepared from WT and *mi/mi*. Images of full‐size immunoblots are shown in Data S3. (d) Oxidized proteins were significantly increased in MA of *mi/mi* (*n* = 8), compared to WT (*n* = 8) (*p* = 0.0001; Student's *t*‐test). (e) Nox2 expression in MA was significantly increased in *mi/mi* (*n* = 8), compared to WT (*n* = 8) (*p* = 0.0019; Student's *t*‐test). Images of full‐size immunoblots are shown in Data S2. (f) Nox4 expression in MA was significantly increased in *mi/mi* (*n* = 8), compared to WT (*n* = 8) (*p* = NS; Student's *t*‐test). Images of full‐size immunoblots are shown in Data S2.

**FIGURE 6:**
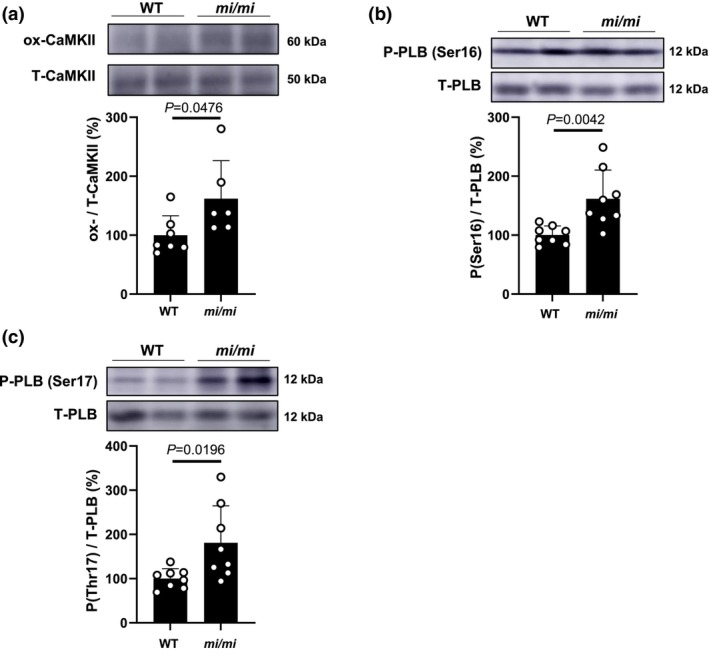
Effects of MITF mutation on expression of ox‐CaMKII and phospho‐PLB in MA. (a) Expression of ox‐CaMKII in MA was significantly increased in *mi/mi* (*n* = 7), compared to WT (*n* = 6) (*p* = 0.0476; Student's *t*‐test). Images of full‐size immunoblots are shown in Data S2. (b) Expression of phospho‐PLB (Ser‐16) in MA was significantly increased in *mi/mi* (*n* = 8), compared to WT (*n* = 8) (*p* = 0.0042; Student *t*‐test). Images of full‐size immunoblots are shown in Data S2. (c) Expression of phospho‐PLB (Thr‐17) in MA was significantly increased in *mi/mi* (*n* = 8), compared to WT (*n* = 8) (*p* = 0.0196; Student's *t*‐test). Images of full‐size immunoblots are shown in Data S2.

**FIGURE 7:**
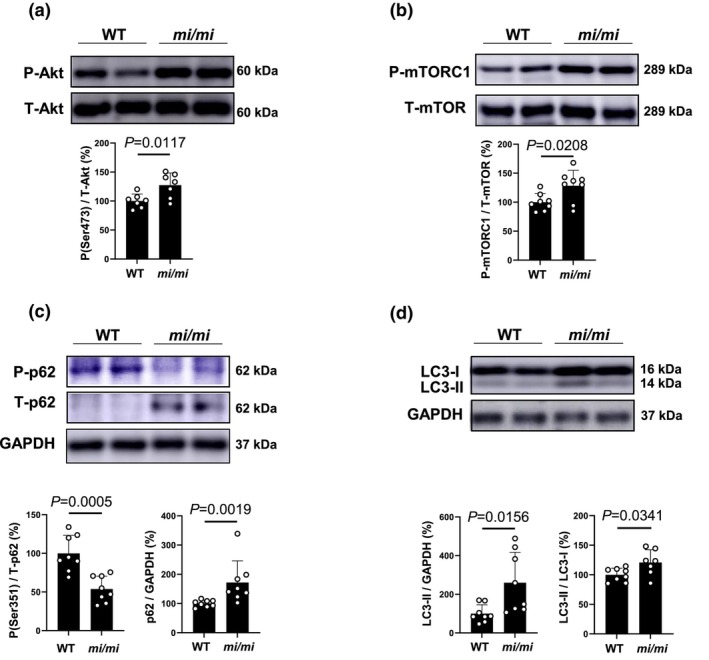
Effects of MITF mutation on expression of phospho‐Akt, phospho‐mTOR, phospho‐p62 and LC3 in MA. (a) Expression of phospho‐Akt (Ser 473) in MA was significantly increased in *mi/mi* (*n* = 7), compared to WT [*n* = 7] (*p* = 0.0117; Student's *t*‐test). Images of full‐size immunoblots are shown in Data S2. (b) Expression of phospho‐mTOR (Ser 2448) in MA was significantly increased in *mi/mi* [*n* = 8], compared to WT [*n* = 8] (*p = 0.0208; Student's *t*‐test). Images of full‐size immunoblots are shown in Data S2. (c) Expression of phosphor‐p62 (Ser 351) (*left*) in MA was significantly decreased in *mi/mi* (*n* = 8), compared to WT (*n* = 8) (*p* = 0.0005 by Student's *t*‐test). However, expression of p62 (*right*) in MA was significantly increased in *mi/mi* (*n* = 8), compared to WT [*n* = 8] (*p* = 0.0019; Mann–Whitney *U* test). Images of full‐size immunoblots are shown in Data S2. (d) LC3‐II/GAPDH (*left*) were significantly increased in *mi/mi* (*n* = 8), compared to WT (*n* = 8) (*p* = 0.0156; Student's *t*‐test). LC3‐II/LC3‐I (*right*) in MA was significantly increased in *mi/mi* (*n* = 7), compared to WT (*n* = 8) (*p* = 0.0341; Student's *t*‐test). Images of full‐size immunoblots are shown in Data S2.

Data are presented as means ± standard deviation (SD). The Shapiro–Wilk test was performed to evaluate if the sample showed a normal distribution (Shapiro & Wilk, 1965) (Data S3). When the distribution was not normal, we used a nonparametric test for analysis. Parametric comparisons were performed using a Student's *t*‐test for 2 groups (Figures 1b, 4a–d, 5b,d–f, 6, 7a–c[left],d; Figures S1a,b, S3d, S4b,d, S5b, S6) or one‐way ANOVA followed by the Tukey–Kramer post hoc test (hereafter abbreviated as ANOVA/Tukey–Kramer) for 3 or more groups (Figures 1c,e, 2d, 3c[left],d[right], 4f). Nonparametric comparisons were performed using a Mann–Whitney *U* test for 2 groups (Figure 7c[right]) or Kruskal–Wallis *H*‐test followed by the Steel‐Dwass post hoc test (hereafter abbreviated as Kruskal–Wallis/Steel‐Dwass) for 3 or more groups (Figures 2b, 3b,c[right],d[left], 4e,g; Figures S2b,d, S3b, S5d). Differences were considered significant when *p* < 0.05.

## 3 RESULTS

### 3.1 Effects of MITF mutation on body weight and MA mass at baseline

Body weight (BW) was significantly decreased in *mi/mi* compared with WT (WT [*n* = 7] vs. *mi/mi* [*n* = 5]: 34 ± 1.6 vs. 21 ± 1.3 g) (*p* < 0.0001; Student's *t*‐test) in accordance with previous findings (Katayama et al., 2010) (Figure 1b).

MA mass was significantly decreased in *mi/mi* compared with WT (WT [*n* = 6] vs. *mi/mi* [*n* = 5]: 136 ± 8.8 vs. 40 ± 7.4) (*p* = 0.0313; ANOVA/Tukey–Kramer) in accordance with previous findings (Katayama et al., 2010; Mori et al., 2013) (Figure 1c).

We also examined tibialis anterior (TA; fast‐switch) mass (Figure S1a) and soleus muscle (SOL; slow‐switch) mass (Figure S1b). TA mass and SOL mass were also significantly decreased in *mi/mi* compared with WT (TA: WT [*n* = 6] vs. *mi/mi* [*n* = 4]: 66 ± 8.9 vs. 45 ± 1.8; *p* = 0.0004, ANOVA/Tukey–Kramer; SOL: WT [*n* = 7] vs. *mi*/*mi* [*n* = 5]: 13 ± 1.3 vs. 9 ± 0.9 g; *p* = 0.0003, ANOVA/Tukey–Kramer).

### 3.2 Effects of MITF mutation on MA mass with/without clenbuterol treatment

We next examined the effects of MITF mutation on β_2_‐AR‐mediated masseter muscle hypertrophy in 3‐week CB‐treated and age‐matched control WT and *mi/mi* (Figure 1c).

MA mass was significantly increased by CB treatment in WT (Control [*n* = 6] vs. CB [*n* = 8]: 136 ± 8.8 vs. 155 ± 16 mg, *p* = 0.0412, ANOVA/Tukey–Kramer), but this increase was suppressed in *mi/mi* (Control [*n* = 5] vs. CB [*n* = 7]: 40 ± 7.4 vs. 51 ± 13 mg, *p* = NS, ANOVA/Tukey–Kramer) (Figure 1c).

We also examined the effects of CB on TA mass (Figure S1a) and SOL mass (Figure S1b). The TA mass was significantly increased by CB treatment in WT (Control [*n* = 6] vs. CB [*n* = 8]: 66 ± 8.9 vs. 77 ± 7.1 mg, *p* = 0.0188, ANOVA/Tukey–Kramer) but the increase was suppressed in *mi/mi* (Control [*n* = 4] vs. CB [*n* = 5]: 45 ± 1.8 vs. 51 ± 1.6 mg, *p* = NS; ANOVA/Tukey–Kramer), as in the case of MA (Figure 1c). In contrast, SOL mass was not increased after CB treatment in WT or *mi/mi*, in accordance with our previous findings (Ito et al., 2019; Ohnuki et al., 2016) (Figure S1b).

These data suggest that β_2_‐AR‐mediated hypertrophic signaling is impaired in *mi/mi*.

### 3.3 Effects of MITF mutation on cross‐sectional area with/without CB treatment

We also examined the CSA, another index of hypertrophy, of MA to confirm the above findings (Figure 1e). CSA of MA was significantly increased by CB treatment in WT (Control [*n* = 7] vs. CB [*n* = 9]: 1898 ± 258 vs. 2754 ± 409 μm^2^, *p* = 0.0080; ANOVA/Tukey–Kramer), but these increases were suppressed in *mi/mi* (Control [*n* = 5] vs. CB [*n* = 6]: 805 ± 19 vs. 966 ± 26.9 μm^2^, *p* = NS; ANOVA/Tukey–Kramer), in accordance with the MA weight data (Figure 1e).

We also examined the CSA of TA (Figure S2a,b) and SOL (Figure S2c,d). CSA of TA was slightly but significantly increased by CB treatment in WT (Control [*n* = 7] vs. CB [*n* = 9]: 3270 ± 88 vs. 3474 ± 94 μm^2^, *p* = 0.0189; Kruskal–Wallis/Steel‐Dwass), but the increase was suppressed in *mi/mi* (Control [*n* = 5] vs. CB [*n* = 6]: 3028 ± 120 vs. 3175 ± 167 μm^2^, *p* = NS; Kruskal–Wallis/Steel‐Dwass) (Figure S2b). In contrast, CSA of SOL was not increased after CB treatment in WT or *mi/mi*, in accordance with our previous findings (Ito et al., 2019; Ohnuki et al., 2016) (Figure S2d).

Thus, the CSA results also support the idea that β_2_‐AR‐mediated hypertrophic signaling is impaired in *mi/mi*.

### 3.4 Effects of MITF mutation on fibrosis in MA

We examined the effects of MITF mutation on MA fibrosis in 3‐week CB‐treated and age‐matched control WT and *mi/mi* by means of Masson‐trichrome staining (Figure 2a). The area of MA fibrosis was similar with/without CB treatment in both WT (Control [*n* = 6] vs. CB [*n* = 9]: 2.6 ± 0.1 vs. 2.6 ± 0.4%, *p* = NS; Kruskal–Wallis/Steel‐Dwass) and *mi/mi* (Control [*n* = 5] vs. CB [*n* = 6]: 7.3 ± 0.9 vs. 8.3 ± 1.4%, *p* = NS; Kruskal–Wallis/Steel‐Dwass). However, the area of fibrosis in MA was approximately 3‐fold greater in both the control and CB‐treated group in *mi/mi*, compared to WT (Control: CTRL WT [*n* = 6] vs. CTRL *mi/mi* [*n* = 5]; *p* = 0.00314; Kruskal–Wallis/Steel‐Dwass; CB WT [*n* = 9] vs. CB *mi/mi* [*n* = 6]; *p* = 0.0079; Kruskal–Wallis/Steel‐Dwass) (Figure 2b). In contrast, the area of fibrosis in TA and SOL was similar with/without CB treatment in both WT and *mi/mi*, and its magnitude was also similar in WT and *mi/mi* (Figure S3).

These data suggest that MA fibrosis is selectively increased in both the control and CB *mi/mi*, while TA and SOL fibrosis are not affected. In addition, β_2_‐AR signaling did not influence the development of MA fibrosis in *mi/mi*.

### 3.5 Effects of MITF mutation on myocyte apoptosis in MA

We next examined the effects of MITF mutation on myocyte apoptosis in MA by means of TUNEL staining (Figure 2c). The level of apoptosis was similar with/without CB treatment in WT (Control [*n* = 6] vs. CB [*n* = 6]: 0.2 ± 0.03 vs. 0.2 ± 0.04%, *p* = NS; ANOVA/Tukey–Kramer). However, TUNEL‐positive MA myocytes in *mi/mi* mice were significantly increased in both the control (WT [*n* = 6] vs. *mi/mi* [*n* = 6]: 0.2 ± 0.03 vs. 30 ± 2.0%, *p* < 0.0001; ANOVA/Tukey–Kramer) and the CB‐treated group (WT [*n* = 6] vs. *mi/mi* [*n* = 6]: 0.2 ± 0.04 vs. 15 ± 1.0%, *p* < 0.0001; ANOVA/Tukey–Kramer), but its magnitude was significantly decreased in CB‐treated *mi/mi* mice (*p* < 0.0001; ANOVA/Tukey–Kramer) (Figure 2d). On the other hand, levels of TUNEL‐positive TA and SOL myocytes were similar with/without CB treatment in both WT and *mi/mi* (Figure S4).

These data suggest that myocyte apoptosis was selectively increased in MA, but not in TA or SOL, in *mi/mi*. In addition, β_2_‐AR stimulation might decrease the level of MA myocyte apoptosis in *mi/mi*.

### 3.6 Effects of MITF mutation on the number of myofibers with centralized nuclei in MA

We also examined the percentage of myofibers with centralized nuclei in MA (Figure 3a), which are associated with apoptosis and skeletal muscle regeneration (Liang et al., 2024). The percentage of MA myofibres with centralized nuclei was similar with/without CB treatment in WT (Control [*n* = 6] vs. CB [*n* = 9]: 0.6 ± 0.5 vs. 0.2 ± 0.4%, *p* = NS; Kruskal–Wallis/Steel‐Dwass). However, the percentage of MA myofibres with centralized nuclei in *mi/mi* mice was significantly increased in both the control (WT [*n* = 6] vs. *mi/mi* [*n* = 5]: 0.6 ± 0.5 vs. 11 ± 1.0%, *p* = 0.0308; Kruskal–Wallis/Steel‐Dwass) and the CB‐treated group (WT [*n* = 9] vs. *mi/mi* [*n* = 6]: 0.2 ± 0.4 vs. 6.9 ± 1.1%, *p* = 0.0056; Kruskal–Wallis/Steel‐Dwass) (Figure 3b). On the other hand, the percentage of myofibers with centralized nuclei in TA and SOL myocytes was similar with/without CB treatment in both WT and *mi/mi* (Figure S5).

These results are consistent with the TUNEL staining data (Figure 2c,d).

### 3.7 Effects of MITF mutation on the expression of caspases 3/9 in MA

The expression levels of full‐length and cleaved caspase‐3 were similar with/without CB treatment in MA of WT (full‐length: Control [*n* = 8] vs. CB [*n* = 8]: 100 ± 4.5 vs. 95 ± 8.5%, *p* = NS; ANOVA/Tukey–Kramer, cleaved: Control [*n* = 8] vs. CB [*n* = 8]: 100 ± 44 vs. 69 ± 36%, *p* = NS; Kruskal–Wallis/Steel‐Dwass). However, the expression levels of full‐length and cleaved caspase‐3 in *mi/mi* mice were significantly increased in both the control (full‐length: WT [*n* = 8] vs. *mi/mi* [*n* = 8]: 100 ± 4.5 vs. 225 ± 30%, *p* < 0.0001; ANOVA/Tukey–Kramer, cleaved: WT [*n* = 8] vs. *mi/mi* [*n* = 8]: 100 ± 44 vs. 2014 ± 1505%, *p* = 0.0043; Kruskal–Wallis/Steel‐Dwass) and the CB‐treated group (WT [*n* = 8] vs. *mi/mi* [*n* = 8]: 95 ± 8.5 vs. 225 ± 24%, *p* < 0.0001; ANOVA/Tukey–Kramer, cleaved: WT [*n* = 8] vs. *mi/mi* [*n* = 8]: 69 ± 36 vs. 1584 ± 761%, *p* = 0.0043; Kruskal–Wallis/Steel‐Dwass) (Figure 3c).

The expression levels of full‐length and cleaved caspase‐9 were similar with/without CB treatment in WT (full‐length: Control [*n* = 8] vs. CB [*n* = 8]: 100 ± 8.1 vs. 88 ± 14%, *p* = NS; Kruskal–Wallis/Steel‐Dwass, cleaved: Control [*n* = 8] vs. CB [*n* = 8]: 100 ± 9.5 vs. 90 ± 19%, *p* = NS; ANOVA/Tukey–Kramer). However, the expressions of full‐length and cleaved caspase‐9 in *mi/mi* mice were significantly increased in both the control (full‐length: WT [*n* = 8] vs. *mi/mi* [*n* = 8]: 100 ± 8.1 vs. 190 ± 33%, *p* = 0.0043; Kruskal–Wallis/Steel‐Dwass, cleaved: WT [*n* = 8] vs. *mi/mi* [*n* = 8]: 100 ± 9.5 vs. 198 ± 43%, *p* < 0.0001; ANOVA/Tukey–Kramer) and the CB‐treated group (full‐length: WT [*n* = 8] vs. *mi/mi* [*n* = 8]: 88 ± 14 vs. 189 ± 36%, *p* = 0.0043; Kruskal–Wallis/Steel‐Dwass, cleaved: WT [*n* = 8] vs. *mi/mi* [*n* = 8]: 90 ± 19 vs. 141 ± 26%, *p* = 0.005; ANOVA/Tukey–Kramer) (Figure 3d).

These results are also consistent with the TUNEL staining data (Figure 2c,d).

### 3.8 Effects of MITF mutation on ERK phosphorylation and α‐SMA expression in MA

We examined the level of phospho‐ERK (Figure 4a) and α‐SMA (Figure 4b) in MA, because these proteins are known to be involved in the development of skeletal muscle fibrosis (Chen, Chen, et al., 2021; Kawamura et al., 2019). Expression of phospho‐ERK (Thr‐202/Tyr‐204) in *mi/mi* was greater than that in WT (WT [*n* = 6] vs. *mi/mi* [*n* = 7]: 98 ± 64 vs. 223 ± 110%, *p* = 0.0319 by Student's *t*‐test) (Figure 4a). Expression of α‐SMA in *mi*/*mi* was also significantly greater than that in WT (WT [*n* = 8] vs. *mi/mi* [*n* = 8]: 100 ± 39 vs. 1106 ± 533%, *p* = 0.0001 by Student's *t*‐test) (Figure 4b).

These results suggest that MA fibrosis in *mi/mi* might be mediated, at least in part, through the activation of ERK signaling and α‐SMA signaling.

### 3.9 Effects of MITF mutation on expression of Bax and Bcl‐2 in MA

We also evaluated MA myocyte apoptosis in *mi/mi* by examining the expression of Bax (Figure 4c), an accelerator of apoptosis, and Bcl‐2 (Figure 4d), a regulator of apoptosis, in masseter muscle (Ito et al., 2019; Muller et al., 2001).

Bax expression was significantly increased (WT [*n* = 8] vs. *mi/mi* [*n* = 8]: 100 ± 13 vs. 184 ± 25%, *p* < 0.0001 by Student's *t*‐test) and Bcl‐2 expression was significantly decreased (WT [*n* = 8] vs. *mi/mi* [*n* = 8]: 100 ± 30 vs. 56 ± 20%, *p* = 0.0042 by Student's *t*‐test).

These data support the idea that MITF mutation might increase TUNEL‐positive MA myocytes at least in part through the increase of Bax expression and decrease of Bcl‐2 expression.

### 3.10 Effects of MITF mutation on miR‐1, miR‐24, and miR‐133 expression in MA

While many miRNAs are expressed ubiquitously, some are expressed in a tissue‐specific manner. Muscle‐specific miR‐1, miR‐206, and miR‐133a are well established to be necessary for proper skeletal muscle development and function (Duran et al., 2015; Sun et al., 2008; Townley‐Tilson et al., 2010), but the effects of MITF mutation on the expression of miR‐1, miR‐206 and miR‐133a in MA have not been reported. We thus examined the expression of miR‐1 (Figure 4e), miR‐206 (Figure 4f) and miR‐133a (Figure 4g) in MA of WT and *mi/mi* at 1, 4 and 12 weeks of age by means of real‐time PCR.

The expression of miR‐1 in MA was gradually increased and reached a maximum at 12 weeks after birth in WT (1 week [*n* = 6] vs. 12 weeks [*n* = 6]: 100 ± 72 vs. 1231 ± 979%, *p* = 0.0456; Kruskal–Wallis/Steel‐Dwass). However, its expression in MA was depressed during the developmental stage of *mi/mi* (Figure 4e).

The expression of miR‐206 in MA reached a maximum at 4 weeks after birth in WT (1 week [*n* = 5] vs. 4 weeks [*n* = 5]: 100 ± 38 vs. 389 ± 154%, *p* < 0.0001: ANOVA/Tukey–Kramer). Its expression in MA was depressed during the developmental stage of *mi/mi* (Figure 4f).

The expression of miR‐133a in MA was depressed during the developmental stage in both WT and *mi/mi* (Figure 4g).

These data suggest that the alteration in growth and development of MA in *mi/mi* is mediated at least in part through the dysregulation of miR‐1 and miR‐206 during development.

### 3.11 Effects of MITF mutation on miR‐24 in MA

miR‐24 is a non‐muscle‐specific miRNA, but plays an important role in skeletal growth and development (Sun et al., 2008). However, the effects of MITF mutation on the miR‐24 expression in MA are unclear. We thus examined the expression of miR‐24 in MA during the developmental stage (Figure S6).

The expression of miR‐24 reached maximum at 8 weeks after birth (1 week [*n* = 4] vs. 8 weeks [*n* = 4]: 100 ± 36 vs. 318 ± 137%, *p* = 0.0018: ANOVA/Tukey–Kramer). In contrast. its expression in *mi/mi* was depressed during the developmental stage, as in the case of muscle‐specific miRNAs.

These data suggest that proper growth and development of MA are altered in *mi/mi* at least in part through the depressed expression of miR‐24, in addition to the depressed expression of muscle‐specific miRNAs such as miR‐1 and miR‐206 during the developmental stage.

### 3.12 Effects of MITF mutation on oxidative stress in MA

Skeletal muscles consume large quantities of oxygen and can generate substantial amounts of reactive oxygen species (ROS) in mitochondria during normal respiration. However, ROS accumulation contributes to skeletal muscle loss and dysfunction. The effects of MITF mutation on ROS generation in MA are not well understood (Harman, 1956). We thus evaluated the effects of MITF mutation on oxidative stress in MA by means of 8‐OHdG immunostaining (Figure 5a,b) and immunoblotting of oxidized proteins (Figure 5c,d).

First, to validate 8‐OHdG immunostaining, we prepared positive and negative control sections by incubating cells with (positive control)/without (negative control) 0.3% H_2_O_2_ in Tris‐buffered saline with Tween 20 (TBS‐T) for 1 h at room temperature before the anti‐8‐OHdG antibody treatment. The results confirmed that the 8‐OHdG staining procedure clearly discriminates 8‐OHdG‐positive and non‐positive nuclei (Figure S7).

The ratio of 8‐OHdG‐positive/total cells was significantly increased in *mi/mi*, compared to the WT control (WT [*n* = 6] vs. *mi/mi* [*n* = 6]: 4.4 ± 0.3 vs. 25 ± 4.1%, *p* < 0.0001 by Student's *t*‐test) (Figure 5b). The amount of oxidized proteins, measured using OxiSelect™, was also significantly increased in *mi/mi*, compared to WT (WT [*n* = 8] vs. *mi/mi* [*n* = 8]: 100 ± 35 vs. 175 ± 22%, *p* = 0.0001 by Student's *t*‐test) (Figure 5d).

These data suggest that ROS production in MA is significantly increased in *mi/mi* and might contribute, at least in part, to the decrease of MA weight and increase of fibrosis and myocyte apoptosis in MA of *mi/mi*.

### 3.13 Effects of MITF mutation on Nox2 and Nox4 expression in MA

ROS is produced through a number of pathways, including Nox, and may be involved in various physiological and pathological processes in skeletal muscle, including fibrosis (Chen, Qian, et al., 2021), myocyte apoptosis (Liu et al., 2022) and muscle weakness (Regan et al., 2017). However, the effects of MITF mutation on the expression of Nox2 and Nox4 in MA are unclear.

So far, it has been demonstrated that two Nox isoforms, Nox2 and Nox4, are expressed in skeletal muscle, and their activity is regulated by their expression level (Kuroda et al., 2010; Zhao et al., 2016). We therefore examined the expression of Nox2 and Nox4 proteins in MA and found that Nox2 protein expression was significantly increased in *mi*/*mi* (WT [*n* = 8] vs. *mi/mi* [*n* = 8]: 100 ± 23 vs. 186 ± 59%, *p* = 0.0019; Student's *t*‐test) (Figure 5e). We also examined Nox4 protein expression and found that it was similar to that in WT and *mi/mi* mice (Figure 5f).

These data suggest that increased ROS production in MA might be derived, at least in part, from the increased Nox2 expression in *mi/mi*.

### 3.14 Effects of MITF mutation on CaMKII oxidation in MA

Recently, it has been shown that the CaMKII can be activated by oxidation of methionine residues at positions 281 and 282 in MA (Ito et al., 2019). Consequently, increased CaMKII‐dependent sarcoplasmic reticulum (SR) calcium leakage might contribute to MA fibrosis and apoptosis (Ito et al., 2019). More importantly, oxidative activation of CaMKII by ROS via Nox2 was recently demonstrated to induce SR Ca^2+^ leakage and sarcopenia in skeletal muscle (Kadoguchi et al., 2018). However, the effects of MITF mutation on the oxidative CaMKII‐mediated activation of SR Ca^2+^ leakage in MA have not been reported. We thus examined the level of ox‐CaMKII in MA and found that it was significantly increased in *mi/mi*, compared with WT (CaMKII: WT [*n* = 7] vs. *mi/mi* [*n* = 6]: 100 ± 33 vs. 162 ± 65%, *p* = 0.0476; Student *t*‐test) (Figure 6a).

These data, taken together with previous findings, suggest that activation of Nox2/CaMKII signaling in MA might induce SR Ca^2+^ leakage, leading to increased fibrosis and myocyte apoptosis in *mi/mi*.

### 3.15 Effects of MITF mutation on PLB phosphorylation in MA

The importance of PLB regulation of SR calcium transport ATPase (SERCA2a) function for cardiac muscle health and disease is well established (Kuschel et al., 1999; Luo et al., 1994). We (Ito et al., 2019) and another group (Valentim et al., 2022) recently showed that it might also play an important role in the development of skeletal muscle disease. However, the effect of MITF mutation on PLB regulation in MA has not been established. We therefore examined the effects of MITF mutation on PLB phosphorylation at serine 16 (Figure 6b) and threonine 17 (Figure 6c) in MA; these sites are phosphorylated by protein kinase A and CaMKII, respectively (Koss & Kranias, 1996).

Phospho‐PLB (Ser‐16) in MA was significantly increased in *mi/mi* (WT [*n* = 8] vs. *mi/mi* [*n* = 8]: 100 ± 15.6 vs. 162 ± 48.7%, *p* = 0.0042; Student's *t*‐test), compared to WT (Figure 6b). In addition, phospho‐PLB (Thr‐17) in MA was significantly increased in *mi/mi*, compared to WT (WT [*n* = 8] vs. *mi/mi* [*n* = 8]: 100 ± 22.4 vs. 181 ± 83.7%, *p* = 0.0196; Student's *t*‐test) (Figure 6c).

These data, together with the previous results, suggest that increased PLB phosphorylation might also cause impairment of Ca^2+^ homeostasis in MA myocytes of *mi/mi*.

### 3.16 Effects of MITF mutation on Akt–mTOR signaling

Autophagy plays a protective role in sarcopenia by relieving oxidative stress in skeletal muscle (Kinoshita et al., 2019; Xie et al., 2023). However, the effects of MITF mutation on autophagy in MA remain unclear. We thus examined the effects of MITF mutation on autophagy in MA.

We first examined the phosphorylation of Akt (Ser 473) and mTOR phosphorylation at serine 2448 (mTORC1) in MA of *mi/mi*, since Akt‐mTORC1 might inhibit autophagy in cells, including skeletal myocytes (Sato et al., 2015). Phosphorylation levels of Akt (Ser‐473) (Figure 7a) and mTORC1 (Ser‐2448) (Figure 7b) were similarly and significantly increased in MA of *mi/mi*, compared with WT (Akt: WT [*n* = 7] vs. *mi/mi* [*n* = 7]: 100 ± 12.0 vs. 127 ± 21.2%, *p* = 0.0017; Student's *t*‐*test*. mTORC1: WT [*n* = 8] vs. *mi/mi* [*n* = 8]: 100 ± 14.7 vs. 128 ± 26.9%, *p* = 0.0208; Student's *t*‐test).

These results suggest that autophagy might be inhibited in MA of *mi/mi* via activation of Akt‐mTORC1 signaling.

### 3.17 Effects of MITF mutation on phosphorylation and expression of p62

Increased p62 phosphorylation on serine 351 is required for autophagic degradation of polyubiquitinated proteins and for recruitment of autophagy machinery proteins (Ishimura et al., 2014). We thus examined the level of p62 expression as well as phosphorylation of serine 351, and found that p62 phosphorylation on serine 351 was significantly decreased in MA of *mi/mi*, compared to WT (WT [*n* = 8] vs. *mi/mi* [*n* = 8]: 100 ± 23.3 vs. 54 ± 16.8%, *p* = 0.0005; Student's *t*‐test) (Figure 7c, left). However, p62 expression was significantly increased in MA of *mi/mi* (WT [*n* = 8] vs. *mi/mi* [*n* = 8]: 100 ± 11.6 vs. 172 ± 74.2%, *p* = 0.0019; Mann–Whitney *U* test) (Figure 7c, right).

These data suggest that autophagic activity in MA was decreased in *mi/mi*.

### 3.18 Effects of MITF mutation on LC3

Increased LC3‐II could reflect either increased autophagosome formation due to autophagy induction, or a blockage in the downstream steps from autophagy, such as insufficient fusion or decreased autophagosome degradation (Ishimura et al., 2014).

We thus examined the amount of LC3‐II in terms of the ratio of LC3‐II/GAPDH and LC3‐II/LC3‐I, which correlates with the number of autophagosomes and provides a good index of autophagy induction (Zhang et al., 2016).

LC3‐II/GAPDH (Figure 7d, left) in MA was significantly increased in *mi/mi* (LC3‐II/GAPDH: WT [*n* = 8] vs. *mi/mi* [*n* = 8]: 100 ± 46 vs. 260 ± 158%, *p* = 0.0156; Student's *t*‐test), as was LC3‐II/LC3‐I (Figure 7d, right) (WT [*n* = 8] vs. *mi/mi* [*n* = 7]: 100 ± 9.2 vs. 117 ± 20%, *p* = 0.0341; Student's *t*‐test).

These data, together with the data shown in Figure 7a,b, suggest that there might be a blockage in the downstream steps from autophagy in MA of *mi/mi*.

## 4 DISCUSSION

Although MITF is expressed in a wide variety of tissues, its expression is relatively higher in skeletal and cardiac muscles (Ooishi et al., 2012), where the major isoforms are MITF‐A and MITF‐H, respectively (Ooishi et al., 2012). However, although masticatory function plays a pivotal role in ensuring good general health and quality of life, the role of MITF in MA remains poorly understood. Furthermore, most previous studies were performed using limb muscles such as TA or SOL (Lahoud et al., 2023). In this study, therefore, we examined the role of MITF in MA using *mi/mi* mutant mice.

Previous work has demonstrated that the ratio of heart weight to body weight is significantly decreased at baseline and the hypertrophic response following β‐adrenergic stimulation with isoproterenol is also decreased in *mi/mi* mutant mice (Tshori et al., 2006). In addition, cardiac function is significantly decreased at baseline and after chronic isoproterenol stimulation (Tshori et al., 2006). In this study, we found that the MA mass was significantly decreased at baseline and the hypertrophic response of MA following β‐adrenergic stimulation with CB was also decreased in *mi/mi* mutant mice. We also found that fibrosis and myocyte apoptosis, major causes of skeletal muscle weakness including MA (Ebert et al., 2019; Ito et al., 2019), were significantly increased at baseline in MA of *mi/mi*. These data suggest that MITF might play a key role in growth and β‐AR‐induced hypertrophy as in the case of cardiac muscle.

The clustering and maintenance of nAChR at high density in the postsynaptic membrane are critical for the efficient transmission of neuronal signals from motor neurons to skeletal muscle (Martinez‐Pena & Akaaboune 2021). We have previously demonstrated that excessive fragmentation and decrease in the volume of nAChR clusters in *mi/mi* are observed specifically in MA, but not in limb muscles (TA and gastrocnemius) (Kota et al., 2009; Mori et al., 2013). We speculated that denervation‐induced disuse atrophy resulting from NMJ instability via altered nAChR clustering might contribute at least in part to the MA atrophy in *mi/mi*.

We speculated that denervation‐induced disuse atrophy might occur predominantly in MA in MITF‐mutant mice. Denervation‐induced disuse atrophy is characterized by a progressive decrease of muscle mass and muscle fibers (i.e., decrease of CSA) (Huang et al., 2024) along with fibrosis (Huang et al., 2024) and myocyte apoptosis (Marzetti et al., 2010), leading to a reduced quality of life for individuals. Recently, denervation‐induced disuse atrophy was demonstrated to induce mitochondrial dysfunction (Marzetti et al., 2010) and lysosomal impairment (Triolo et al., 2022), leading to stimulation of mitochondria‐mediated apoptosis (i.e., increase of Bax and decrease of Bcl‐2), ROS signaling (i.e., increase of Nox2 expression) and autophagy flux (i.e., increase of p62 phosphorylation and LC3‐II) (Powers et al., 2012; Triolo et al., 2022). In addition, Ca^2+^ homeostasis might be altered by denervation‐induced disuse atrophy, leading to atrophy of muscle fibers, decreased skeletal muscle performance, and even death (Monti et al., 2021). Ca^2+^ homeostasis also induces CaMKII activation and regulates the phosphorylation status of numerous proteins, including PLB (Ser‐17). Most of these changes were also seen in MA of *mi/mi* in this study (Ingalls et al., 1999; Ito et al., 2019).

Many previous studies have demonstrated that muscle‐specific miR‐1, miR‐133a and miR‐206 are necessary for proper skeletal muscle development and function (Townley‐Tilson et al., 2010). miR‐1, found in both skeletal and cardiac muscle, and miR‐206, specific for skeletal muscle, are known to promote myoblast‐to‐myotube differentiation, while miR‐133 promotes the proliferation of myoblasts and inhibits their differentiation (Townley‐Tilson et al., 2010). Importantly, dysregulation of muscle‐specific miRNA was demonstrated to induce not only cardiac muscle remodeling and heart failure (Carè et al., 2007; Hodgkinson et al., 2015; Sayed et al., 2007; Tatsuguchi et al., 2007), but also skeletal muscle remodeling and diseases (Boettger et al., 2014; Williams et al., 2009). We thus anticipated that the expression of muscle‐specific miR‐1, miR‐133, and miR‐206 might be altered in MA of *mi/mi* during development, leading to decreased muscle volume and muscle remodeling.

We found that the expression of miR‐1, miR‐206 and miR‐24 was increased during the developmental stage in MA of WT. However, their expression was suppressed in MA of *mi/mi*, consistent with our expectation that the decreased expression of miR‐1, miR‐206 and miR‐24 might play a role, at least in part, in the development of MA atrophy and remodeling.

In skeletal muscle cells, Nox2 co‐localizes with membrane protein and acts as the main source of skeletal muscle ROS through the activation of Akt–mTOR signaling (Ferreira & Laitano, 2016; Zhang et al., 2018). In this study, we showed that Akt/mTOR/Nox2 signaling was activated in MA of *mi/mi*, indicating that ROS generation and consequent activation of Akt/mTOR/Nox2 signaling might contribute at least in part to the development of MA atrophy in *mi/mi*.

Recent studies have revealed that autophagy plays a critical role in restoring/maintaining skeletal muscle mass and function under normal conditions, as well as during regeneration and aging, because autophagy is a key homeostatic process, necessary for degrading unwanted cellular components and providing new protein and energy sources (You & Chen, 2021). Importantly, MITF was demonstrated to increase the number of autophagosomes in melanoma cell line or ARPE‐19 cell line, a human retinal pigment epithelial cell line (Martina et al., 2014; Möller et al., 2019). In this study, we found that Akt‐mTORC1 signaling, which might inhibit autophagy in cells, including skeletal myocytes (Sato et al., 2015), was significantly increased in MA of *mi/mi*. The LC3‐II/GAPDH and LC3‐II/LC3‐I ratios, which correlate with the number of autophagosomes, were significantly increased in MA of *mi/mi* (Mizushima et al., 2010). p62 protein was significantly increased and p62 phosphorylation on serine 351, an indicator of autophagy flux, was significantly decreased in MA of *mi/mi* (Bjørkøy et al., 2009; Ishimura et al., 2014). These data, together with our results in this study, suggest that decreased autophagy activity in MA of *mi/mi* might contribute to the decreased MA volume and remodeling.

## 5 LIMITATIONS

Future study will be needed to understand the precise mechanisms through which loss of MITF‐A expression in MA leads to masticatory dysfunction.

## 6 CONCLUSION

Our results support the idea that MITF mutation is associated with MA atrophy, remodeling and dysfunction, and furthermore may lead to suppression of the expression of miRNAs, such as miR‐1, miR‐206 and miR‐24 in MA. Further work is needed to better understand how to target this pathway for the treatment of MA diseases.

## AUTHOR CONTRIBUTIONS

**Megumi Nariyama**, **Yoshiki Ohnuki**, **Satoshi Okumura**: Conceptualization. **Megumi Nariyama**, **Yoshiki Ohnuki**, **Kenji Suita**, **Misao Ishikawa**, **Ichiro Matsuo**, **Takao Mitsubayashi**: Formal analysis.**Megumi Nariyama**, **Ren Matsubara**, **Yasumasa Mototani**, **Aiko Ito**, **Mariko Abe**, **Yoshio Hayakawa**, **Takako Nomura**, **Satoshi Wada**: Investigation. **Megumi Nariyama**, **Yoshiki Ohnuki**, **Kenji Suita**, **Ichiro Matsuo**, **Aiko Ito**: Methodology. **Megumi Nariyama**, **YYoshiki Ohnuki**, **Kenji Suita**, **Ichiro Matsuo**, **Aiko Ito**, **Satoshi Okumura**: Funding acquisition. **Yoshinobu Asada**, **Satoshi Okumura**: Supervision. **Megumi Nariyama**, **Satoshi Okumura**: Writing original draft.

## FUNDING INFORMATION

This study was supported by the Japan Society for the Promotion of Science. (JSPS) KAKENHI Grant (25K13259 and 22K10255 to Megumi Nariyama, 23K09517 to Yoshiki Ohnuki, 23K09493 to Kenji Suita, 24K20123 to Ichiro Matsuo, 24K20067 to Aiko Ito and 24K13250 to Satoshi Okumura). The founders had no role in study design, data collection and analysis, decision to publish, or preparation of the manuscript.

## CONFLICT OF INTEREST STATEMENT

The authors declare that they have no conflict of interest.

## ETHICS STATEMENT

All animal experiments complied with the ARRIVE guidelines (Kilkenny et al., 2009) and were carried out in accordance with the National Institutes of Health guide for the care and use of laboratory animals, as well as institutional guidelines (National Research Council Committee for the Update of the Guide for the Care and Use of Laboratory Animals, 2011). The experimental protocol was approved by the Animal Care and Use Committee of Tsurumi University (No. 22A045).

## Supporting information


Data S1.

Data S2.

Data S3.

## Data Availability

The authors confirm that all data supporting the findings of this study are available within the article and its Supplementary Materials.
