# Supplementary material for: Transcription factor MITF regulates masseter muscle growth and development
Source: Physiol Rep. 2025 Nov 24;13(22):e70677. doi: 10.14814/phy2.70677 (PMC12641285; doi:10.14814/phy2.70677)
Supplement: Supplementary file 1 — Data S1. [file PHY2-13-e70677-s001.pdf]

Data S1

Transcription factor MITF regulates masseter muscle growth and development

Megumi Nariyama [1], Yoshiki Ohnuki [2], Kenji Suita [2], Misao Ishikawa [3], Ren Matsubara [1, 2], Ichiro Matsuo [4], Takao Mitsubayashi [2], Yasumasa Mototani [2], Aiko Ito [6], Mariko Abe [2, 6], Yoshio Hayakawa [7], Satoshi Wada [8], Yoshinobu Asada [1], Satoshi Okumura [2]

[1] Department of Pediatric Dentistry, Tsurumi University School of Dental Medicine, Yokohama 230-8501, Japan
[2] Department of Physiology, Tsurumi University School of Dental Medicine, Yokohama 230-8501, Japan
[3] Department of Oral Anatomy, Tsurumi University School of Dental Medicine, Yokohama 230-8501, Japan
[4] Department of Oral and Maxillofacial Surgery, Ibaraki Medical Center Tokyo Medical University, Ibaraki 300-0395, Japan
[5] Department of Periodontology, Tsurumi University School of Dental Medicine, Yokohama 230-8501, Japan
[6] Department of Orthodontics, Tsurumi University School of Dental Medicine, Yokohama 236-8501, Japan
[7] Department of Dental Anesthesiology, Tsurumi University School of Dental Medicine, Yokohama 230-8501, Japan
[8] Department of Oral and Maxillofacial Facial Surgery, School of Medicine, Kanazawa Medical University, Uchinada, Ishikawa 920-0293, Japan

Figure S1

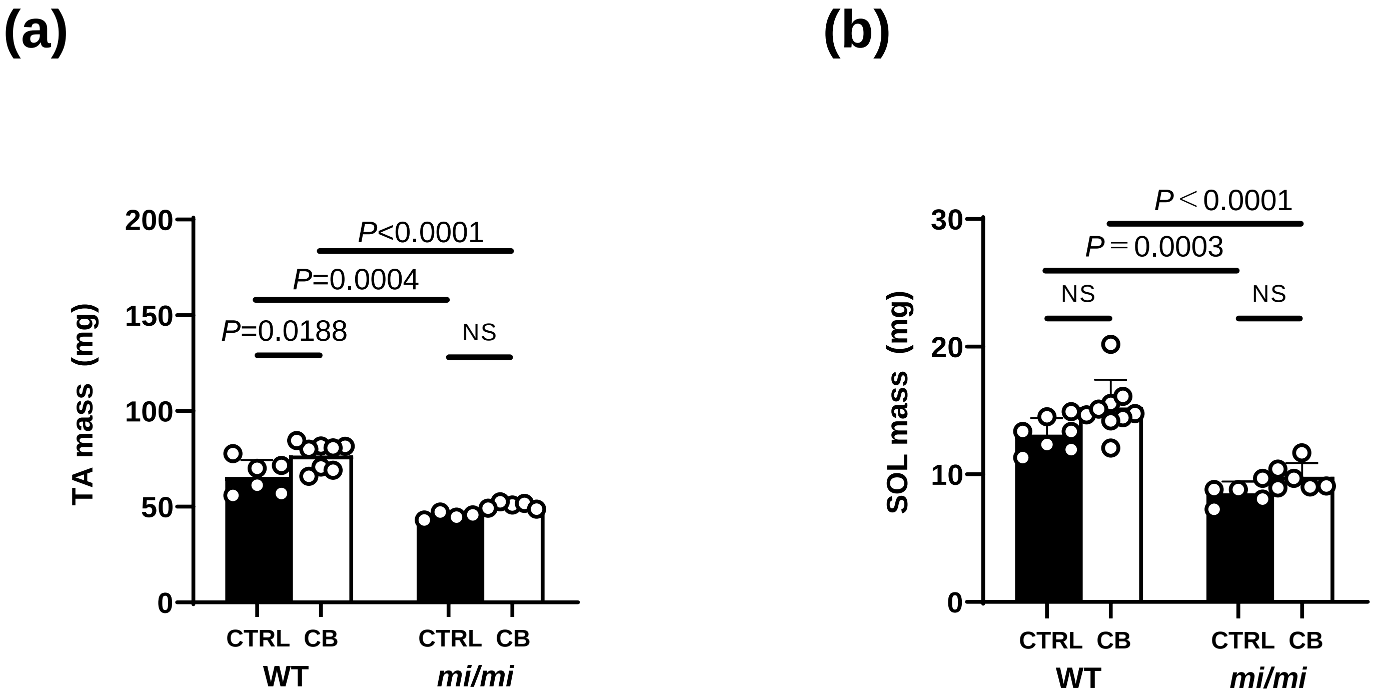

Figure S1

Effects of MITF mutation on TA weight and SOL weight at baseline and after CB treatment.

(a) Tibialis anterior mass was significantly increased by CB treatment in WT (CTRL WT [*n* = 6] vs. CB WT [*n* = 8], *P* = 0.0188; ANOVA/Tukey-Kramer) but the increase was suppressed in *mi/mi* (CTRL *mi/mi* [*n* = 4] vs. CB *mi/mi* [*n* = 5], *P* = NS; ANOVA/Tukey-Kramer).

(b) Soleus muscle mass was similar in both WT and *mi/mi* with/without CB treatment (CTRL WT [*n* = 7] vs. CB WT [*n* = 9], CTRL *mi/mi* [*n* = 5] vs. CB *mi/mi* [*n* = 6], *P* = NS each; ANOVA/Tukey-Kramer).

Figure S2

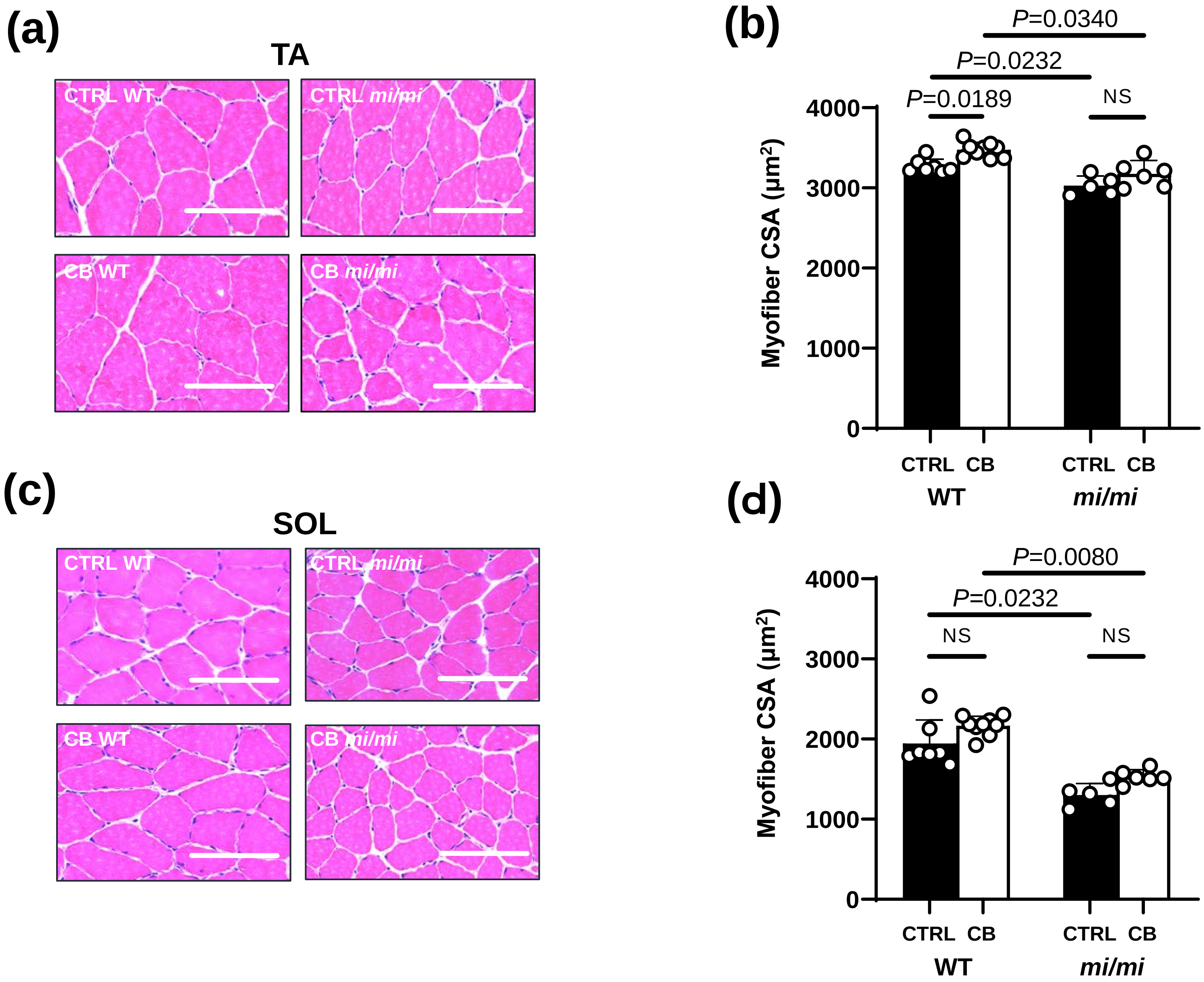

Effects of MITF mutation on cross-sectional area in TA and SOL.

(a-b) Representative images of hematoxylin-eosin-stained sections of TA **(a)**. The CSA was significantly increased by CB treatment in WT but the increase was suppressed in *mi/mi* (Kruskal-Wallis/Steel-Dwass) **(b)**.
(c-d) Representative images of hematoxylin-eosin stained sections of SOL **(c)**. The CSA was not significantly increased by CB treatment in either WT or *mi/mi* (Kruskal-Wallis/Steel-Dwass) **(d)**.

Figure S3

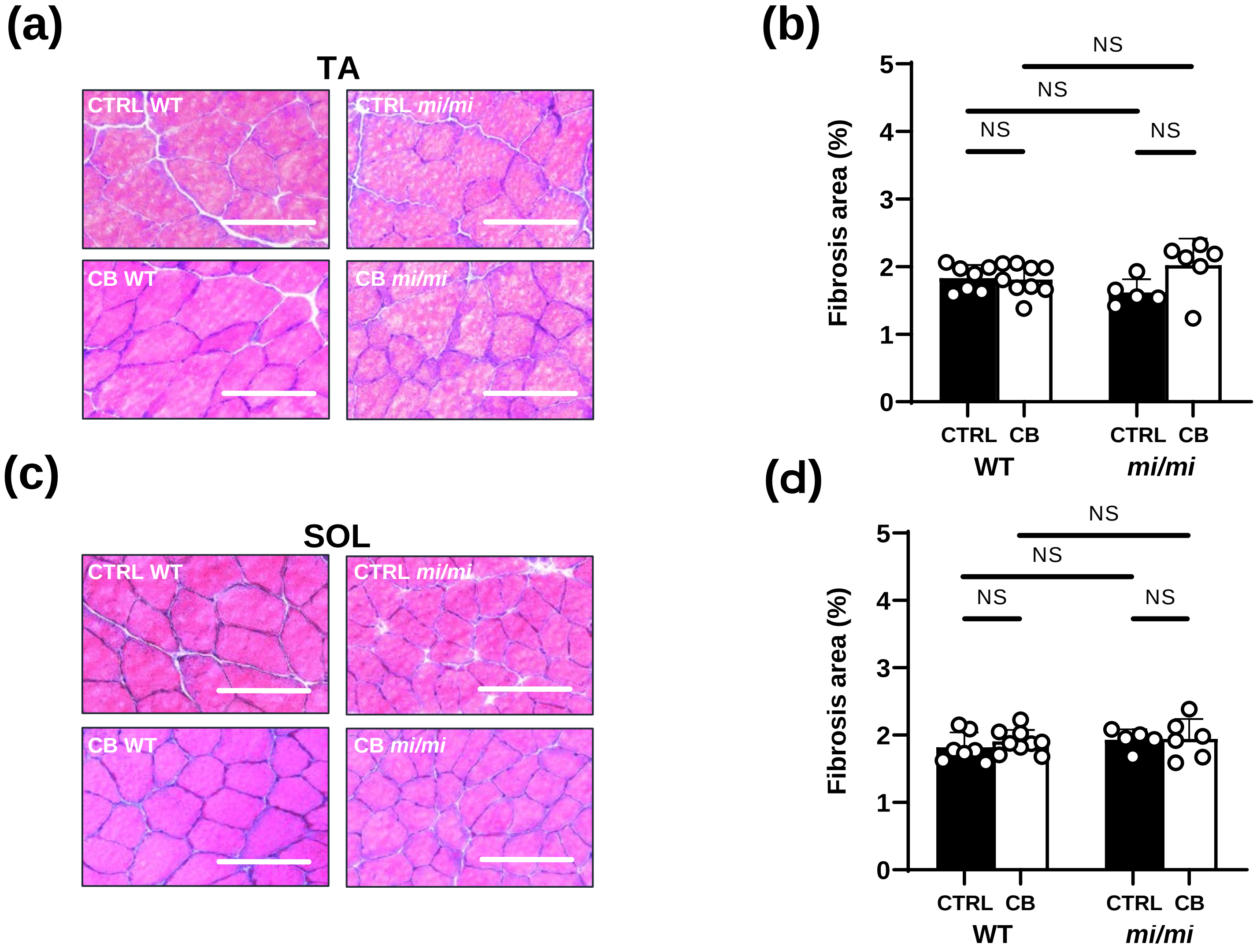

Figure S3

Effects of MITF mutation on area of fibrosis area in MA, TA and SOL.

(a-b) Representative images of Masson-trichrome-stained sections of TA **(a)**. The area of fibrosis was similar among the four groups (Kruskal-Wallis/Steel-Dwass) **(b)**.

(c-d) Representative images of Masson-trichrome-stained sections of SOL **(c)**. The area of fibrosis was similar among the four groups (ANOVA/Tukey-Krame) **(d)**.

Figure S4

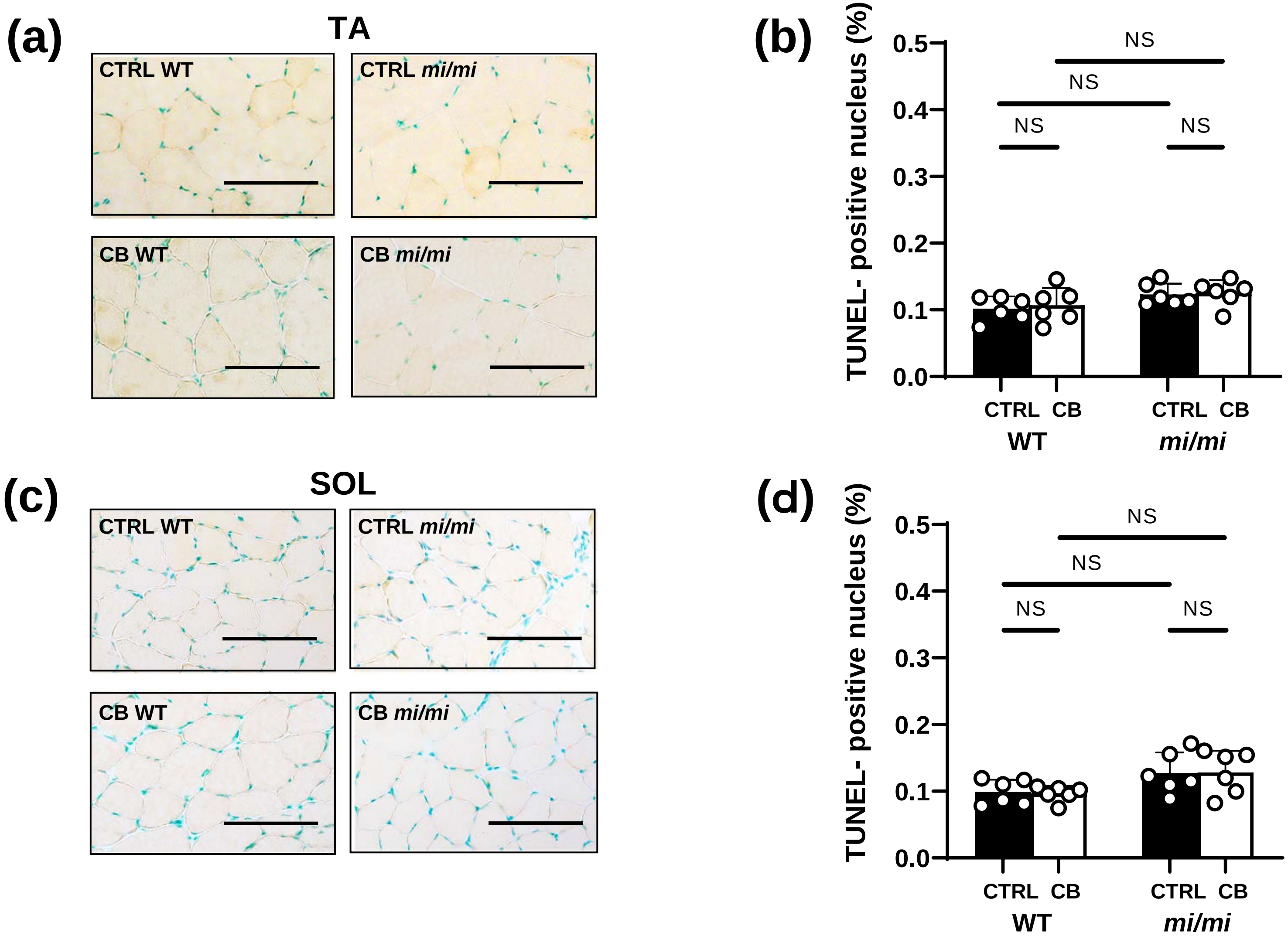

Figure S4

Effects of MITF mutation on myocyte apoptosis in TA and SOL.

(a and b) Representative images of TUNEL-stained sections of TA **(a)**. The number of TUNEL-positive myocytes was similar among the four groups (ANOVA/Tukey-Kramer) **(b)**.

(c and d) Representative images of TUNEL-stained sections of SOL **(c)**. The number of TUNEL-positive myocytes was similar among the four groups (ANOVA/Tukey-Kramer) **(d)**.

Figure S5

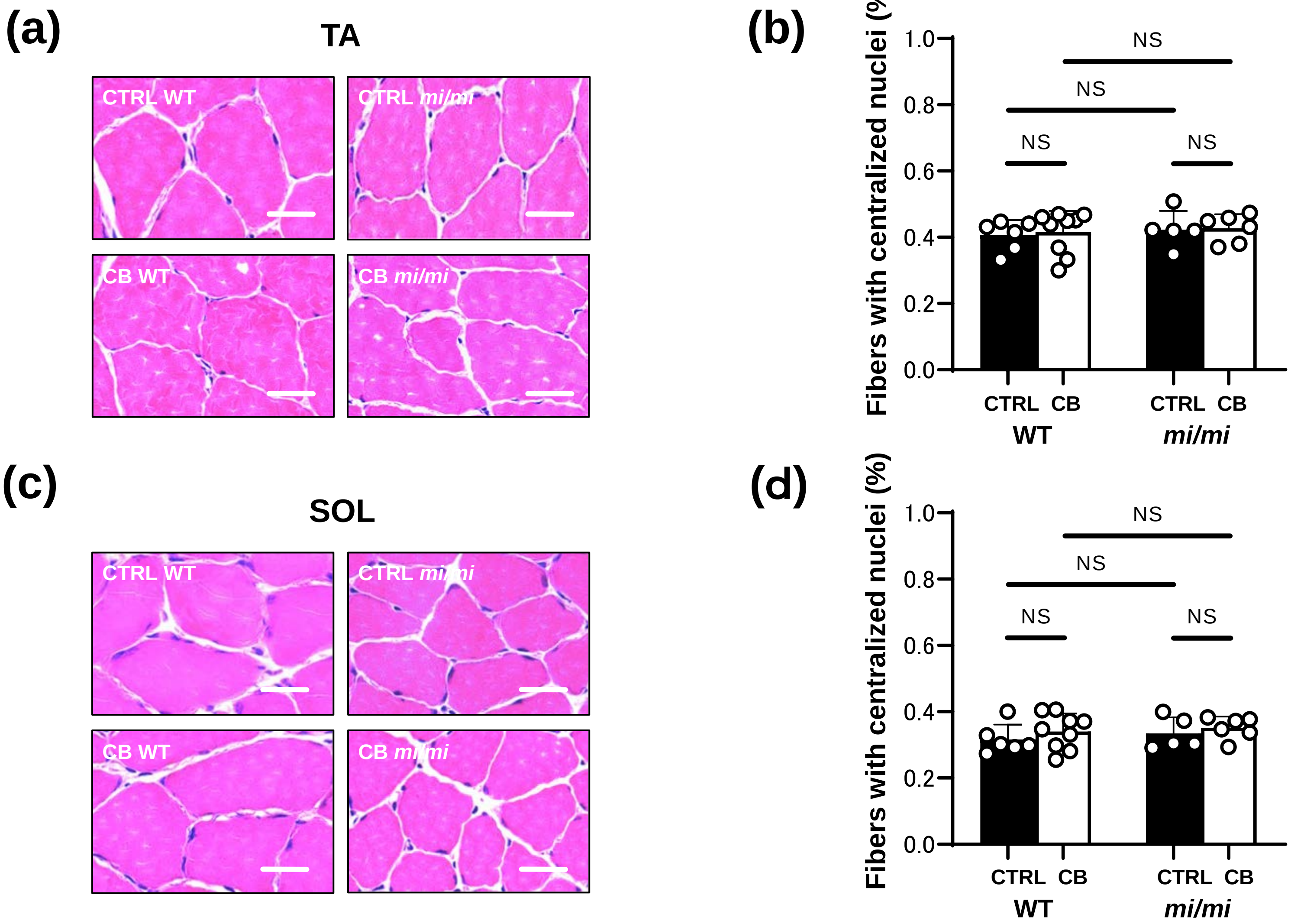

Figure S5

Effects of MITF mutation on the number of myofibers with centralized nuclei in TA and SOL

(a and b) Representative images of hematoxylin-eosin-stained sections of TA **(a)**. The number of myofibers with centralized nuclei was similar among the four groups (ANOVA/Tukey-Kramer) **(b)**. **(c and d)** Representative images of hematoxylin-eosin-stained sections of SOL **(c)**. The number of myofibers with centralized nuclei was similar among the four groups (ANOVA/Tukey-Kramer) **(d)**.

Figure S6

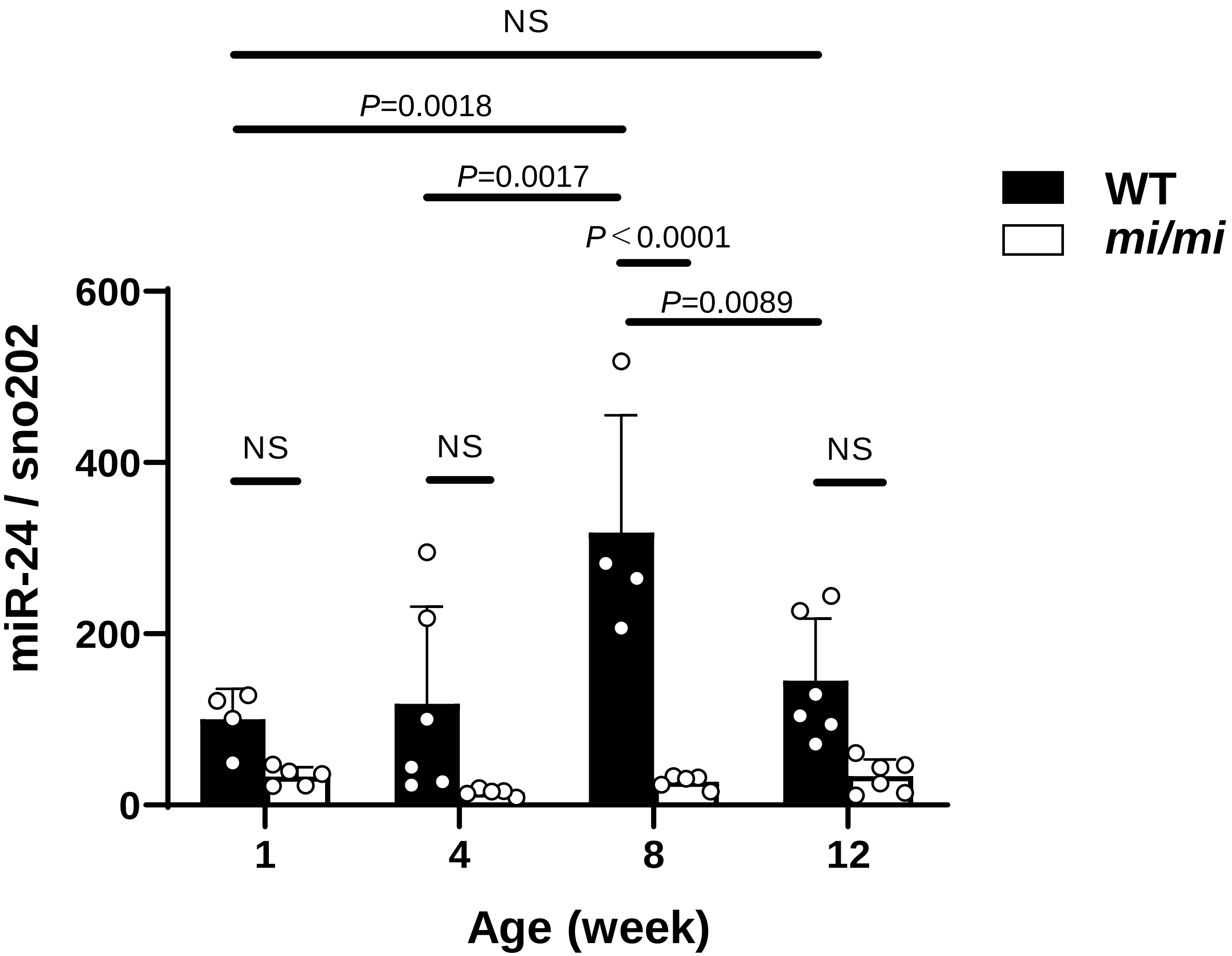

Figure S6

Effects of MITF mutation on expression of miR-24 in MA.

Expression of miR-24 in MA was similar in WT (*n* = 4) and mi/mi (*n* = 5) at 1 week after birth (*P* = NS; ANOVA/Tukey-Kramer). It was significantly increased at 8 weeks after birth in WT (*n* = 4) (*P* = 0.0018; ANOVA/Tukey-Kramer), buy this increase was suppressed in *mi/mi* (*n* = 5).

Figure S7

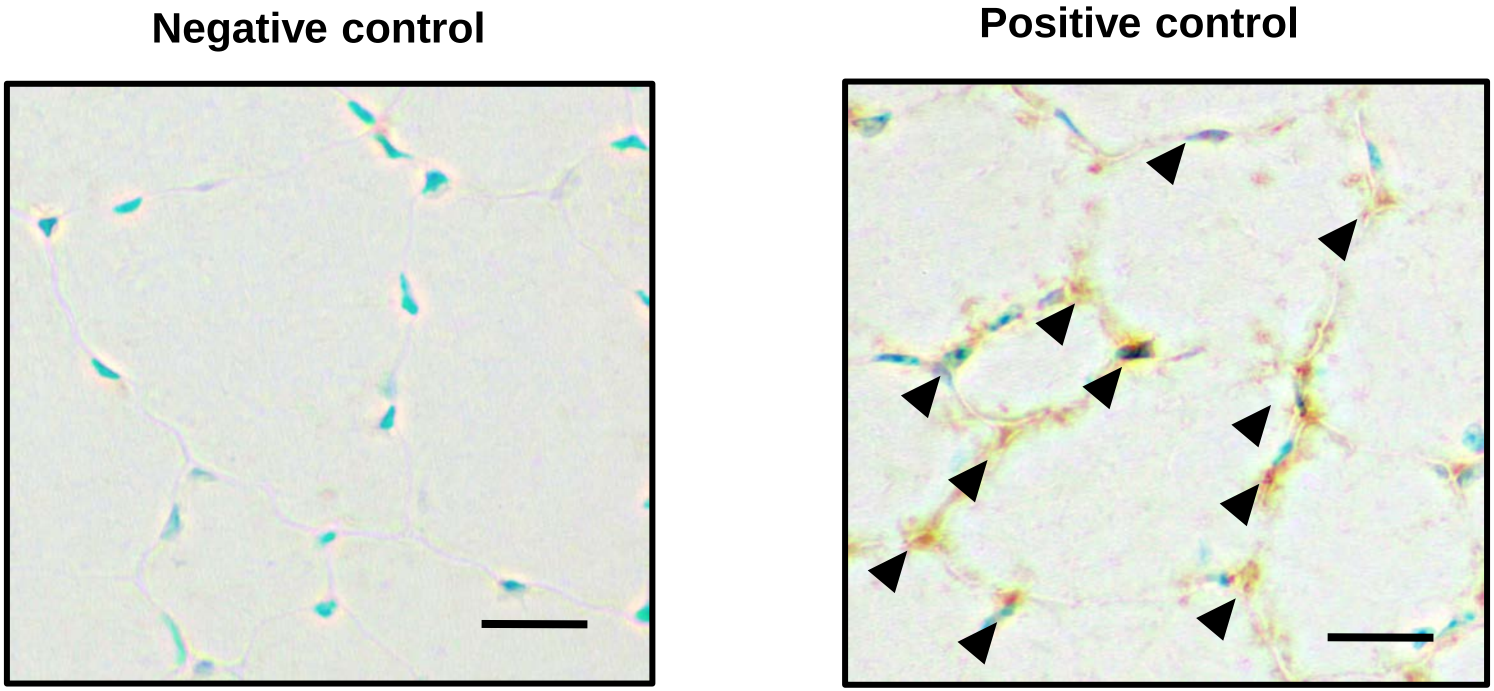

Figure S7

Representative images of negative (*left*) and positive (*right*) controls for 8-OHdG immunostaining. Arrowheads indicates positive immunostaining. Scale bars: 10 μm
