# Supplementary material for: Transcription factor MITF regulates masseter muscle growth and development
Source: Physiol Rep. 2025 Nov 24;13(22):e70677. doi: 10.14814/phy2.70677 (PMC12641285; doi:10.14814/phy2.70677)

Data S2 (Original Images)

Transcription factor MITF regulates masseter muscle growth and development

Megumi Nariyama [1], Yoshiki Ohnuki [2], Kenji Suita [2], Misao Ishikawa [3], Ren Matsubara [1, 2], Ichiro Matsuo [4], Takao Mitsubayashi [2], Yasumasa Mototani [2], Aiko Ito [6], Mariko Abe [2, 6], Yoshio Hayakawa [7], Satoshi Wada [8], Yoshinobu Asada [1], Satoshi Okumura [2]

[1] Department of Pediatric Dentistry, Tsurumi University School of Dental Medicine, Yokohama 230-8501, Japan
[2] Department of Physiology, Tsurumi University School of Dental Medicine, Yokohama 230-8501, Japan
[3] Department of Oral Anatomy, Tsurumi University School of Dental Medicine, Yokohama 230-8501, Japan
[4] Department of Oral and Maxillofacial Surgery, Ibaraki Medical Center Tokyo Medical University, Ibaraki 300-0395, Japan
[5] Department of Periodontology, Tsurumi University School of Dental Medicine, Yokohama 230-8501, Japan
[6] Department of Orthodontics, Tsurumi University School of Dental Medicine, Yokohama 236-8501, Japan
[7] Department of Dental Anesthesiology, Tsurumi University School of Dental Medicine, Yokohama 230-8501, Japan
[8] Department of Oral and Maxillofacial Facial Surgery, School of Medicine, Kanazawa Medical University, Uchinada, Ishikawa 920-0293, Japan

Uncropped western blots used for figure 3 (c)

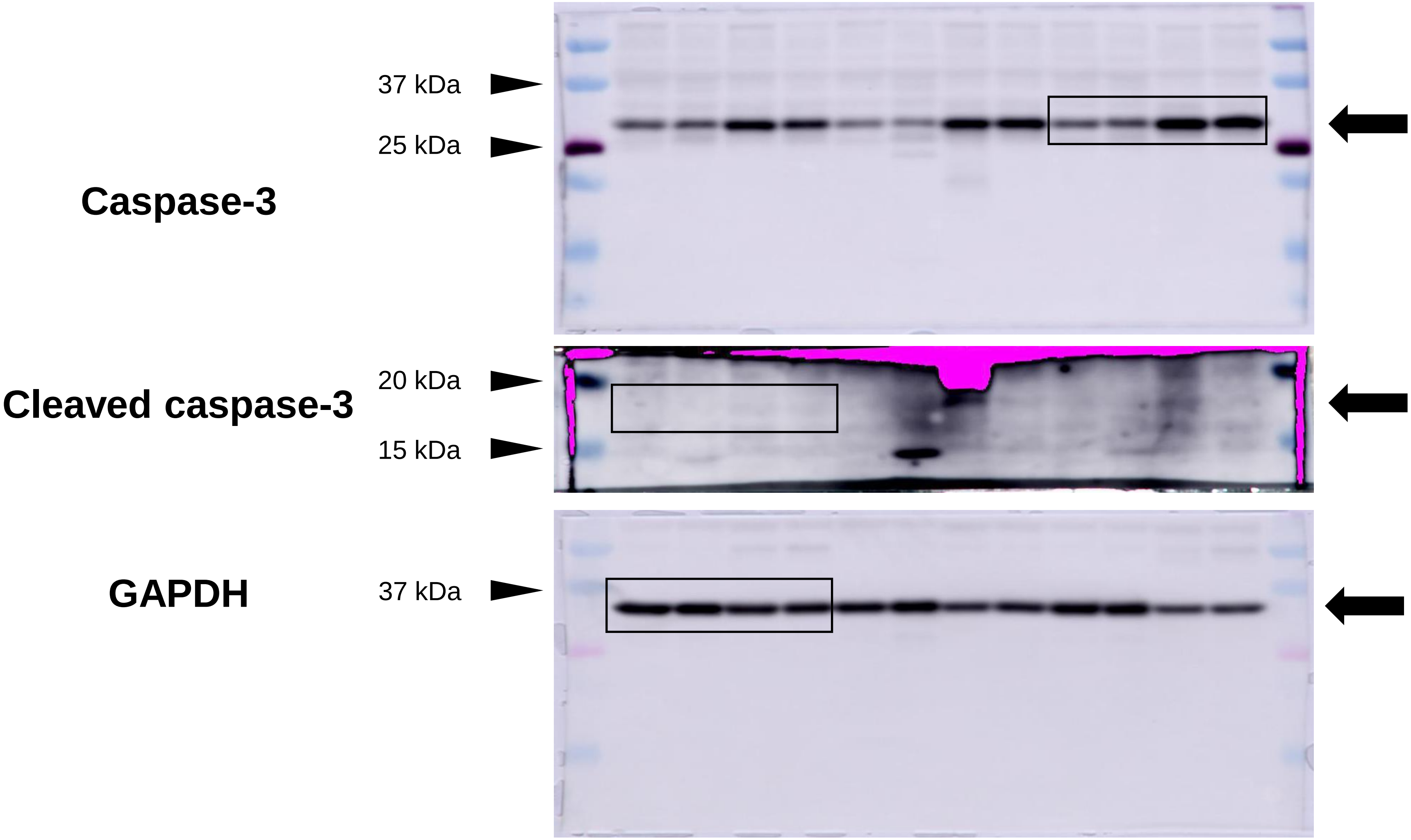

Uncropped western blots used for figure 3 (d)

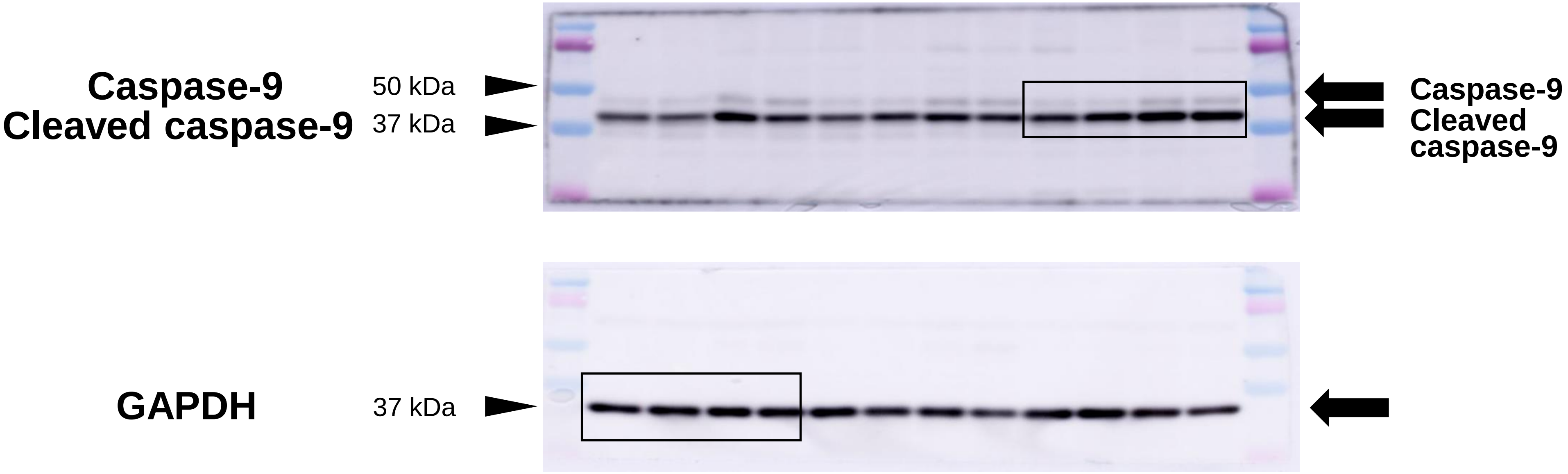

Uncropped western blots used for figure 4 (a)

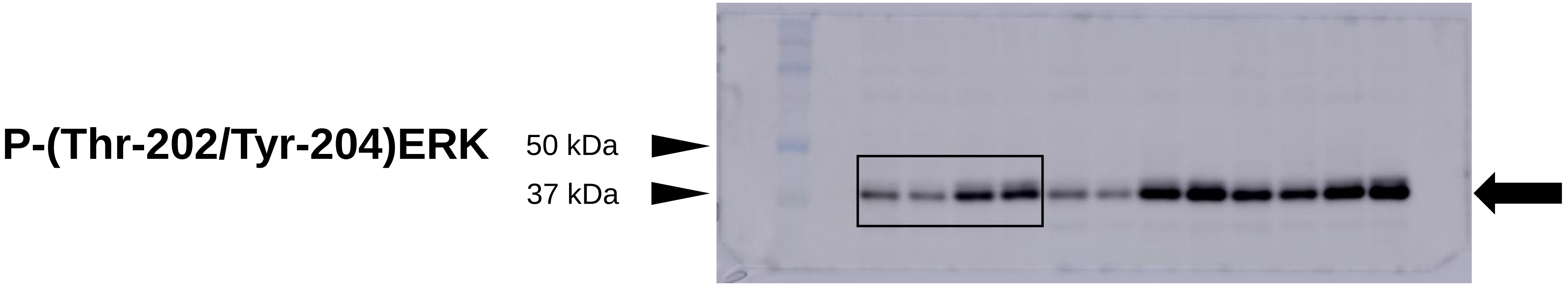

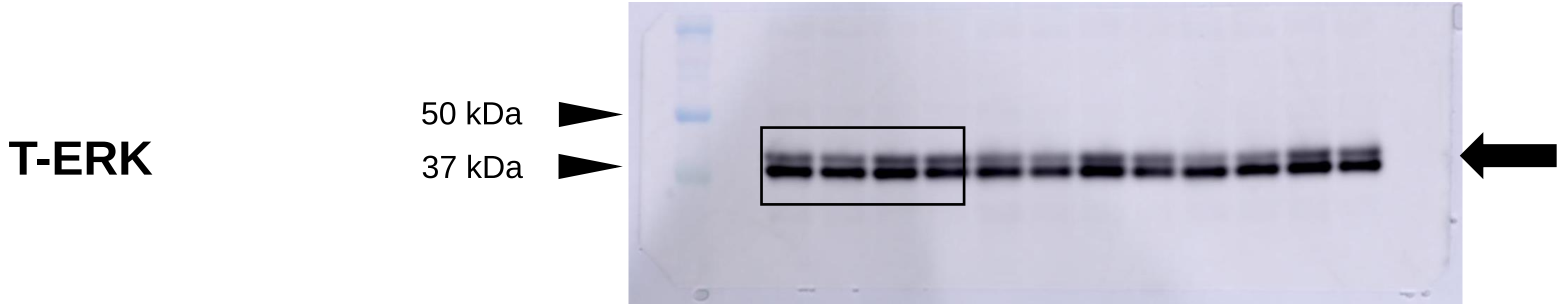

Uncropped western blots used for figure 4 (b)

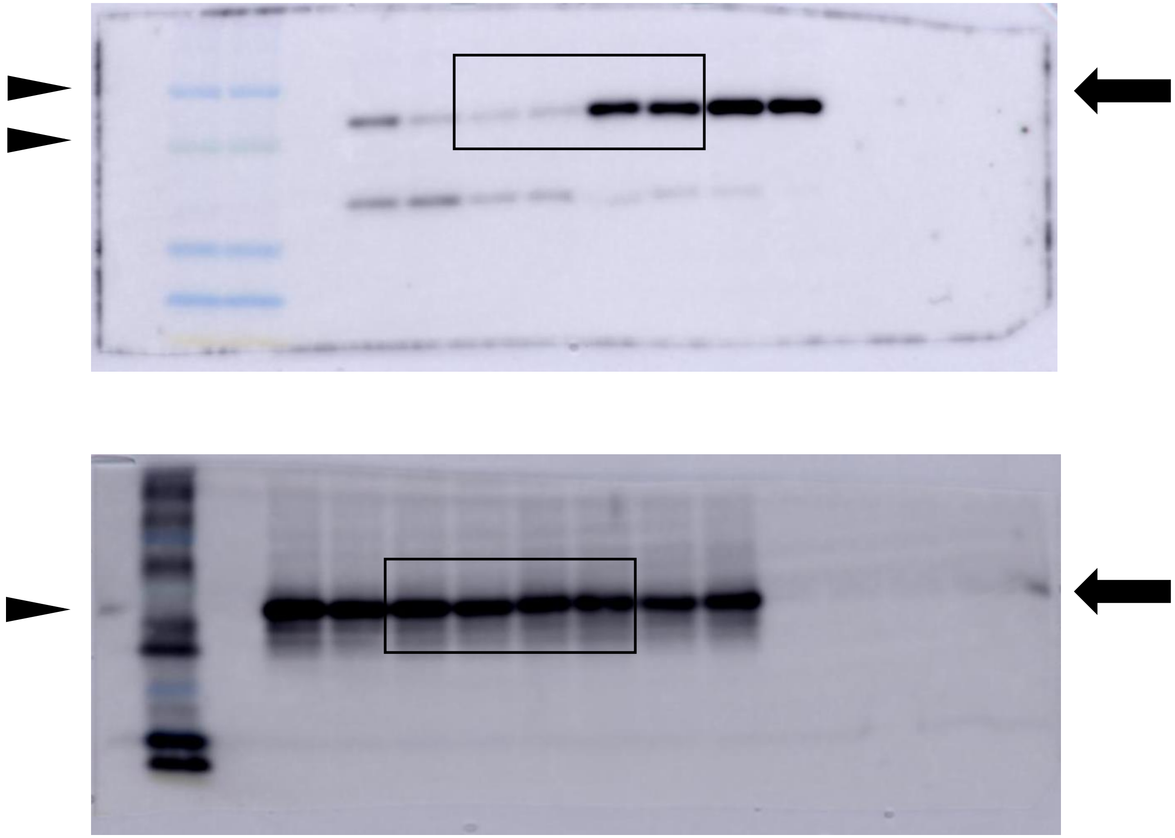

Uncropped western blots used for figure 4 (c)

BAX

37 kDa
25 kDa
20 kDa
15 kDa

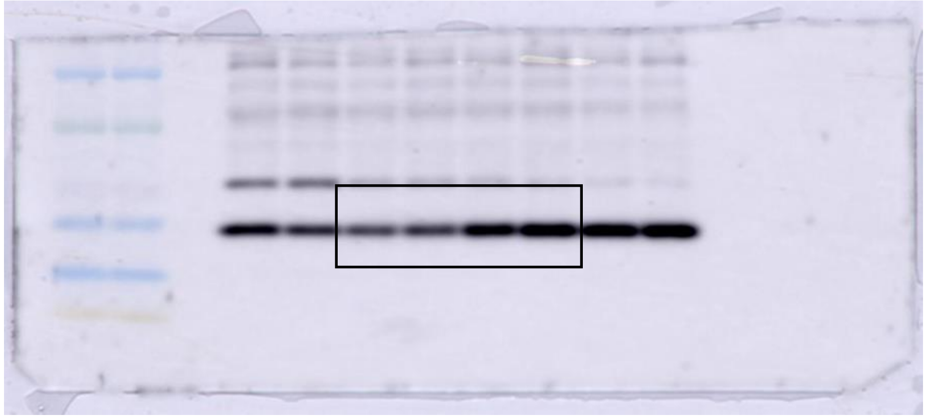

GAPDH

50 kDa
37 kDa
25 kDa

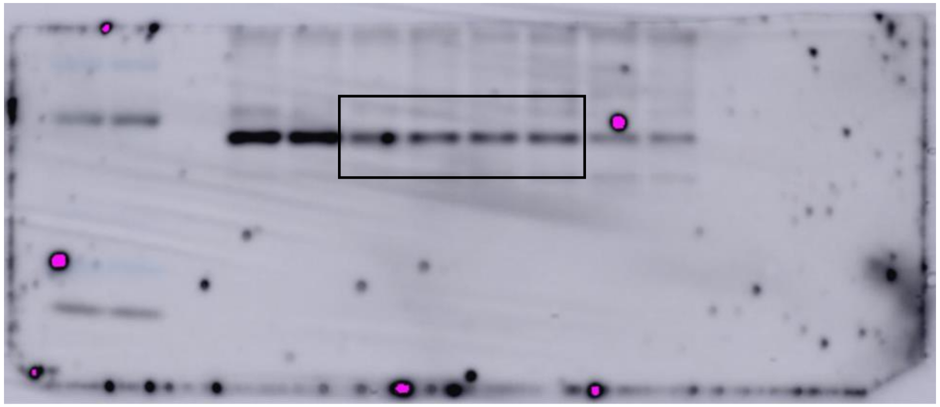

Uncropped western blots used for figure 4 (d)

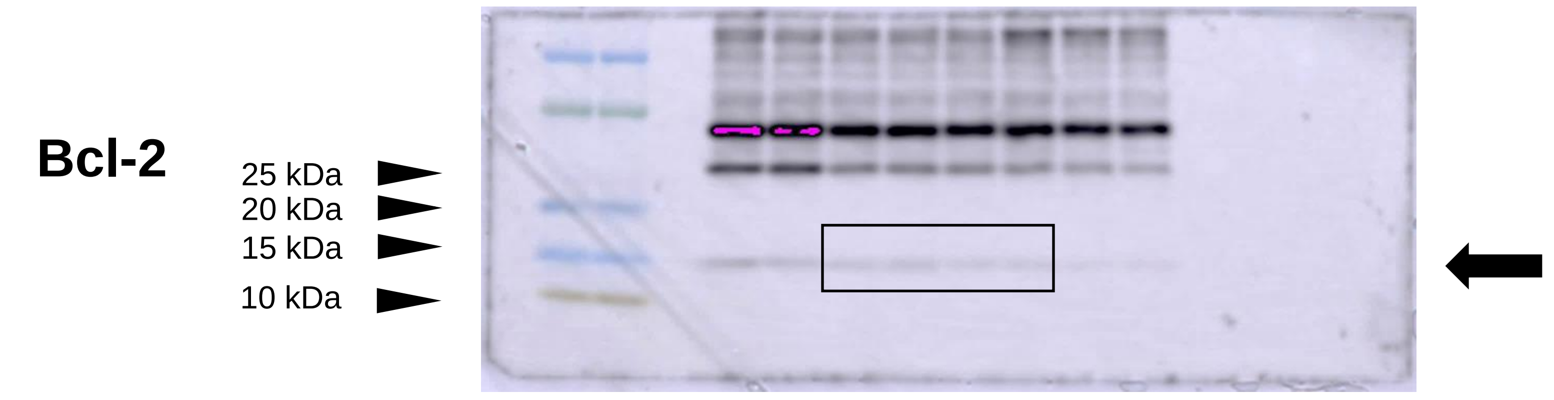

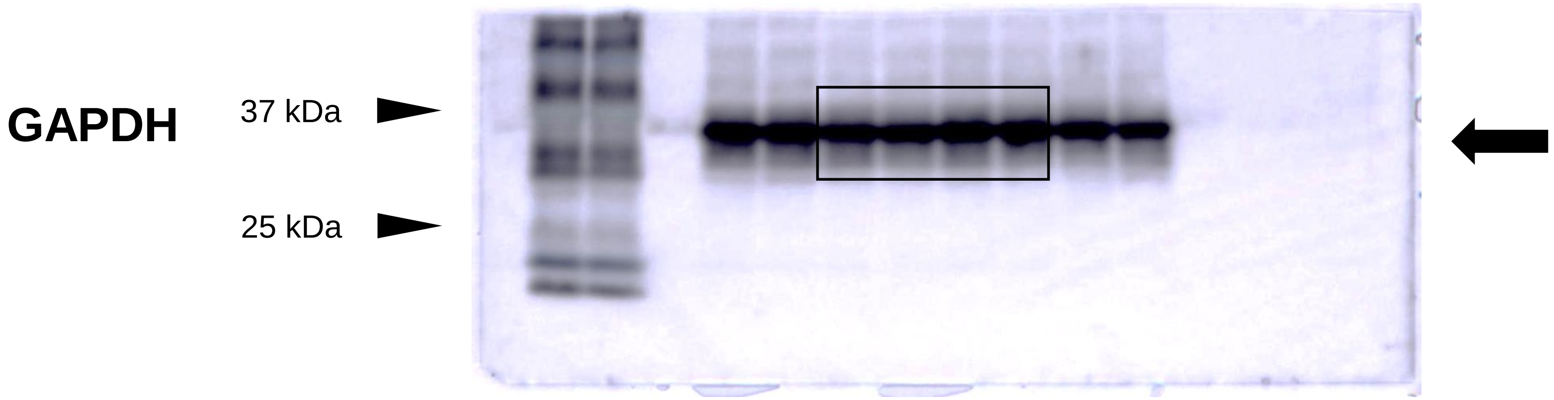

Uncropped western blots used for figure 5 (c)

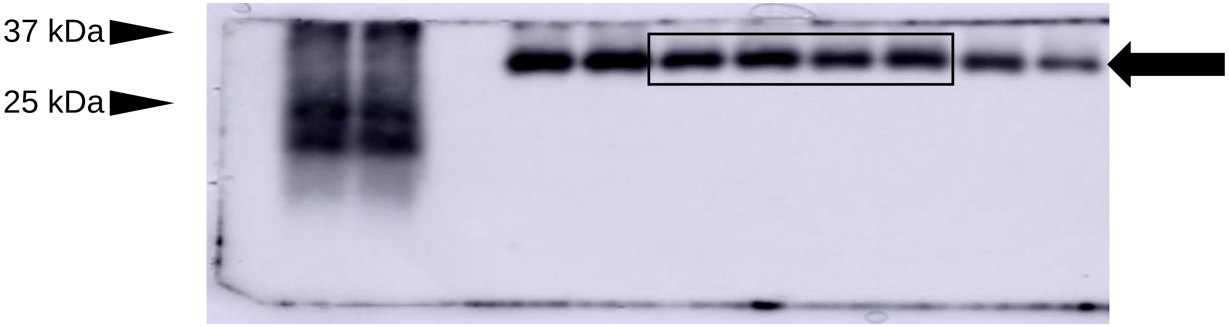

GAPDH

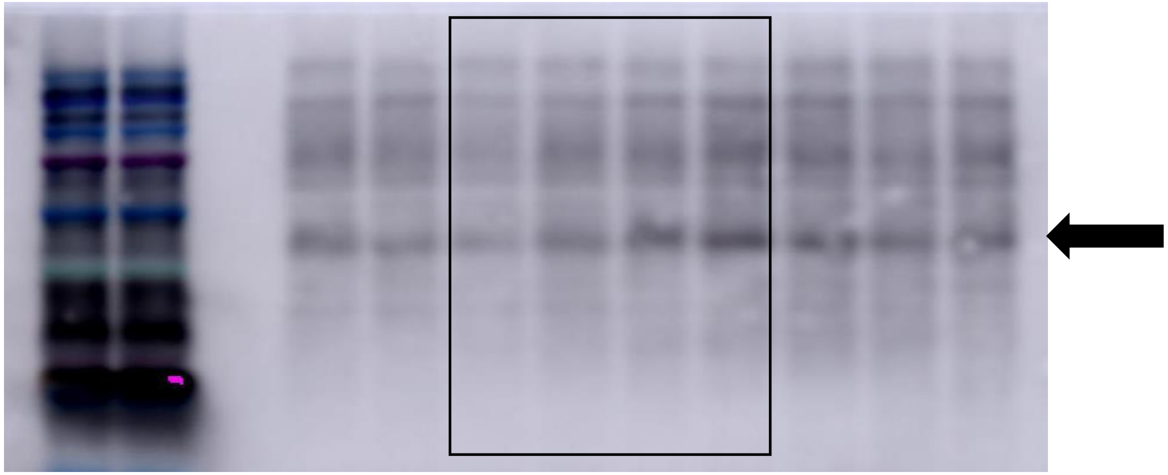

Oxidized protein

Uncropped western blots used for figure 5 (e)

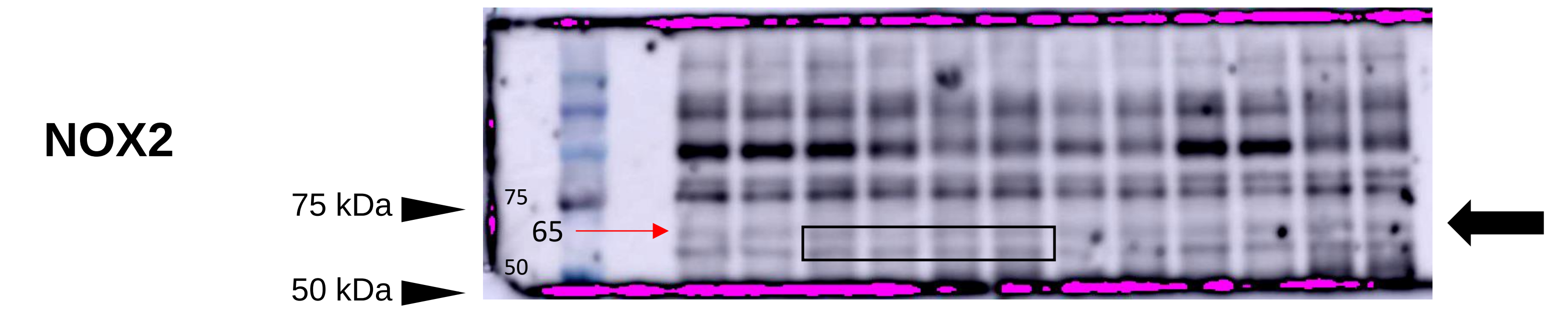

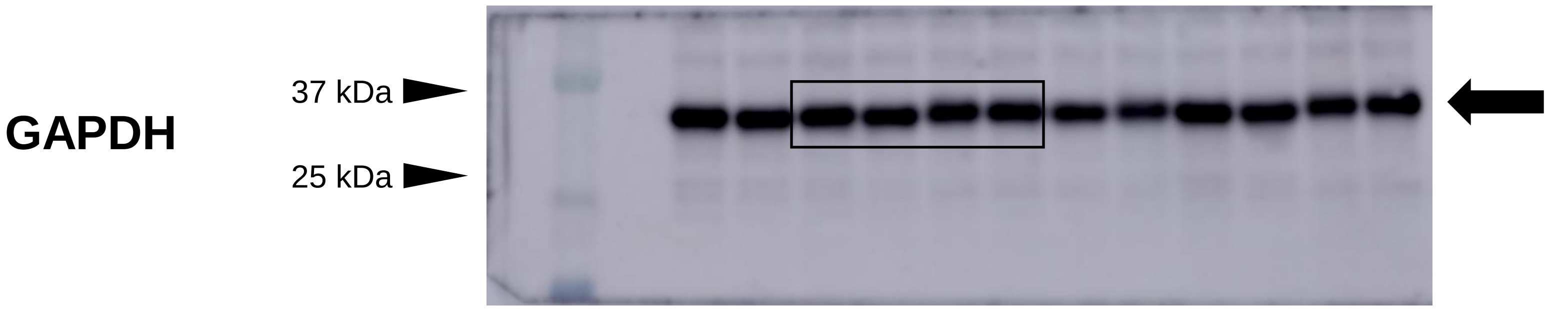

Uncropped western blots used for figure 5 (f)

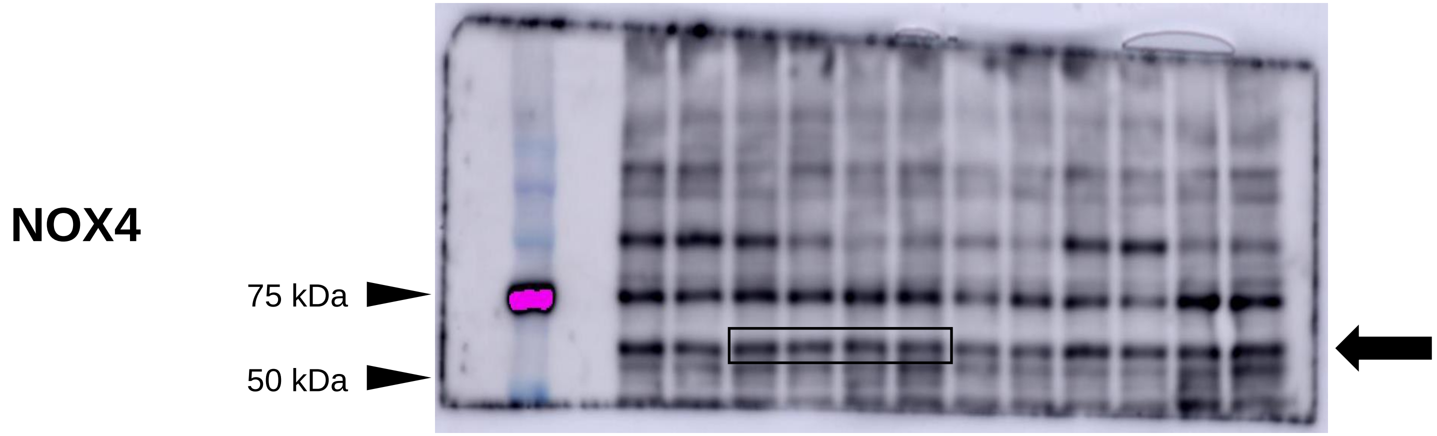

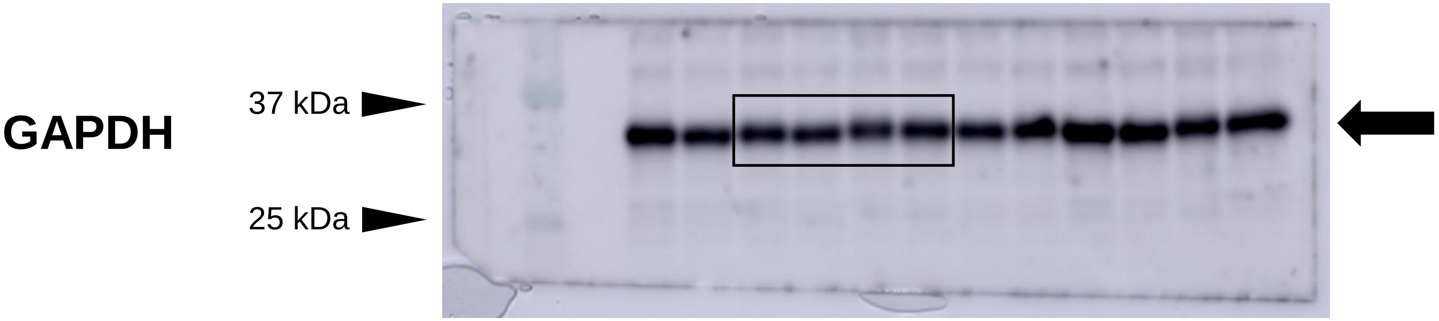

Uncropped western blots used for figure 6 (a)

ox (Met-281/282) -CaMKII

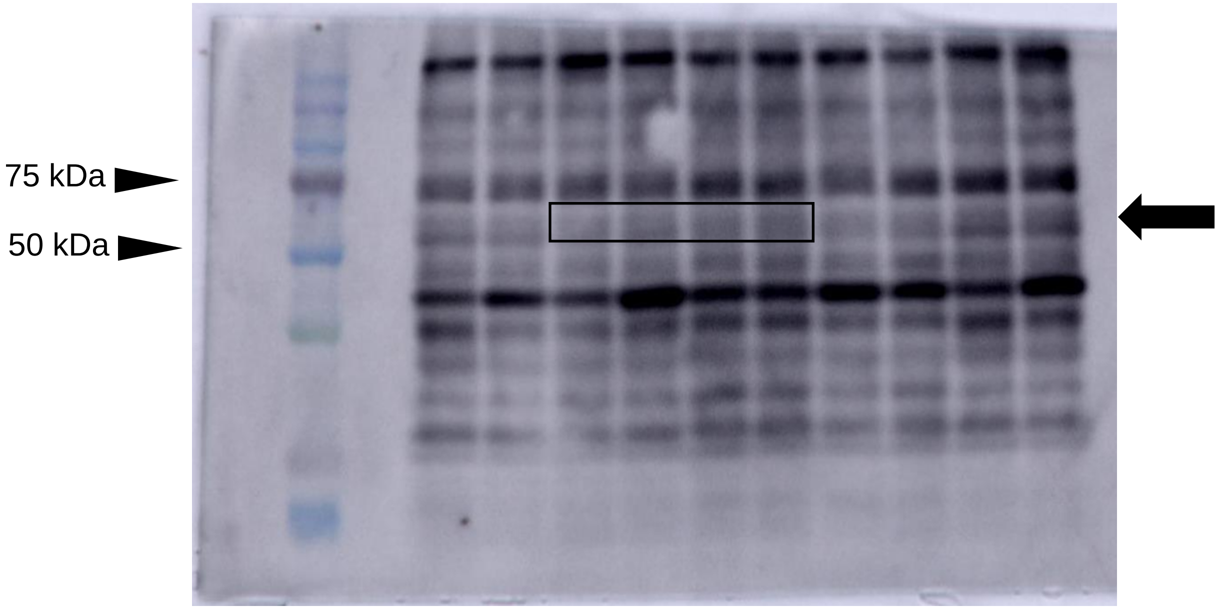

T-CaMKII

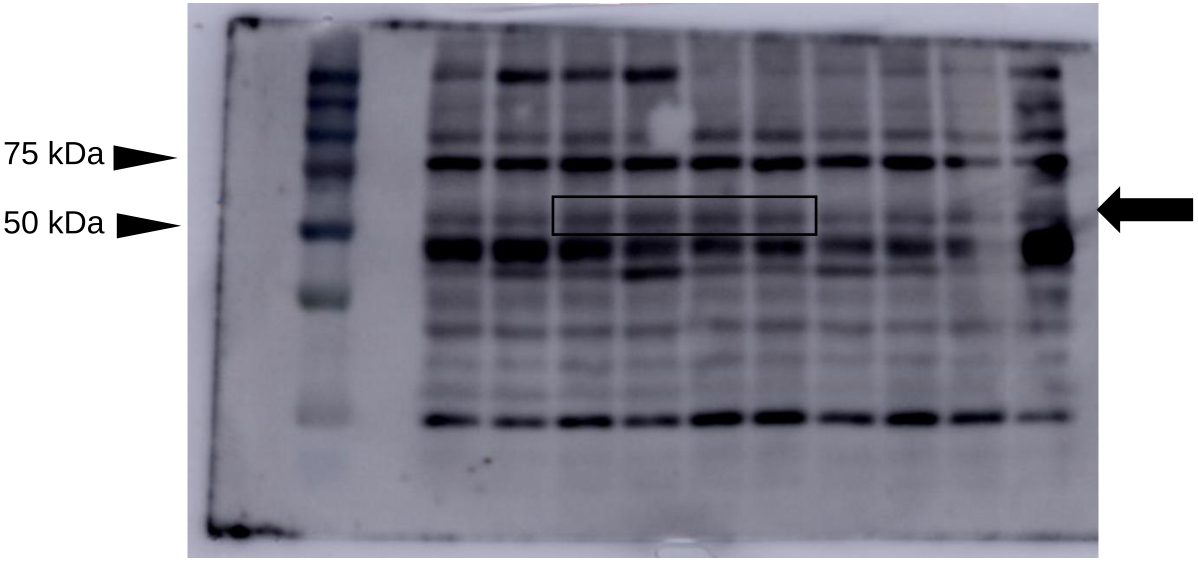

Uncropped western blots used for figure 6 (b)

P(Ser16)-PLB

15 kDa
10 kDa

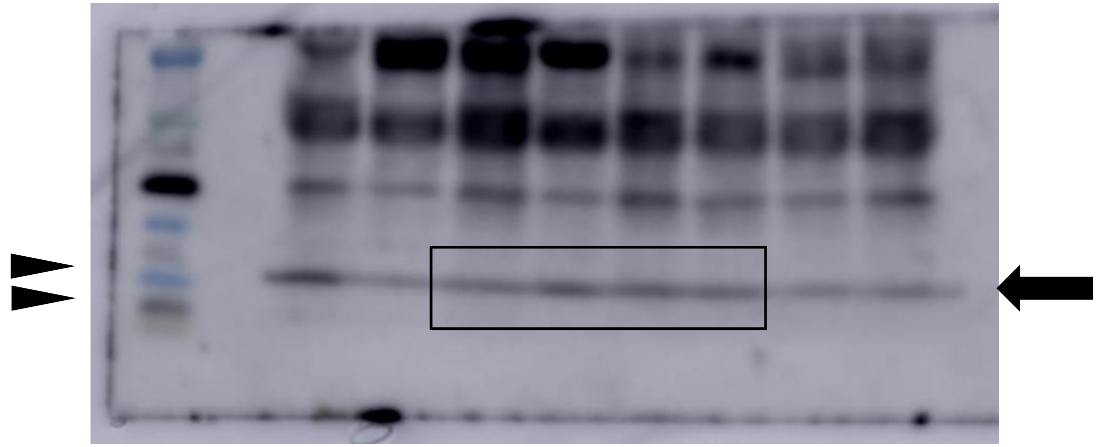

T-PLB

15 kDa
10 kDa

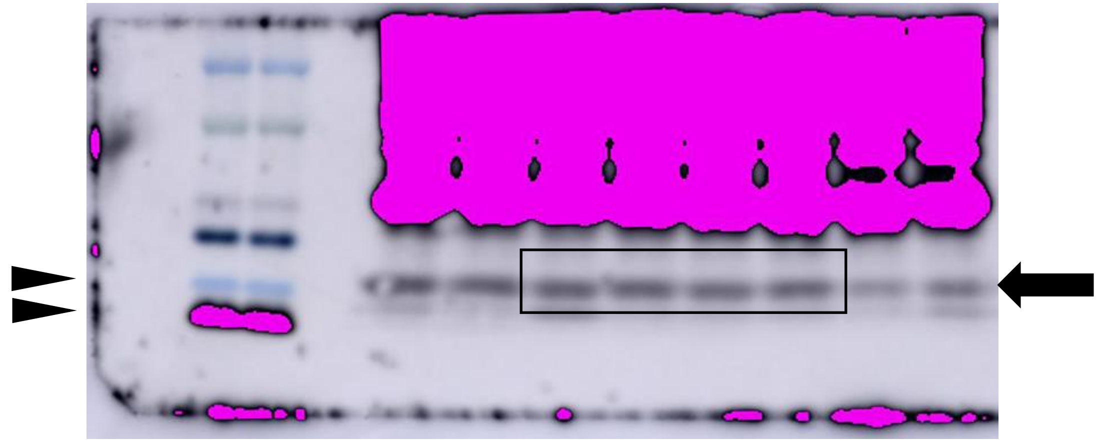

Uncropped western blots used for figure 6 (c)

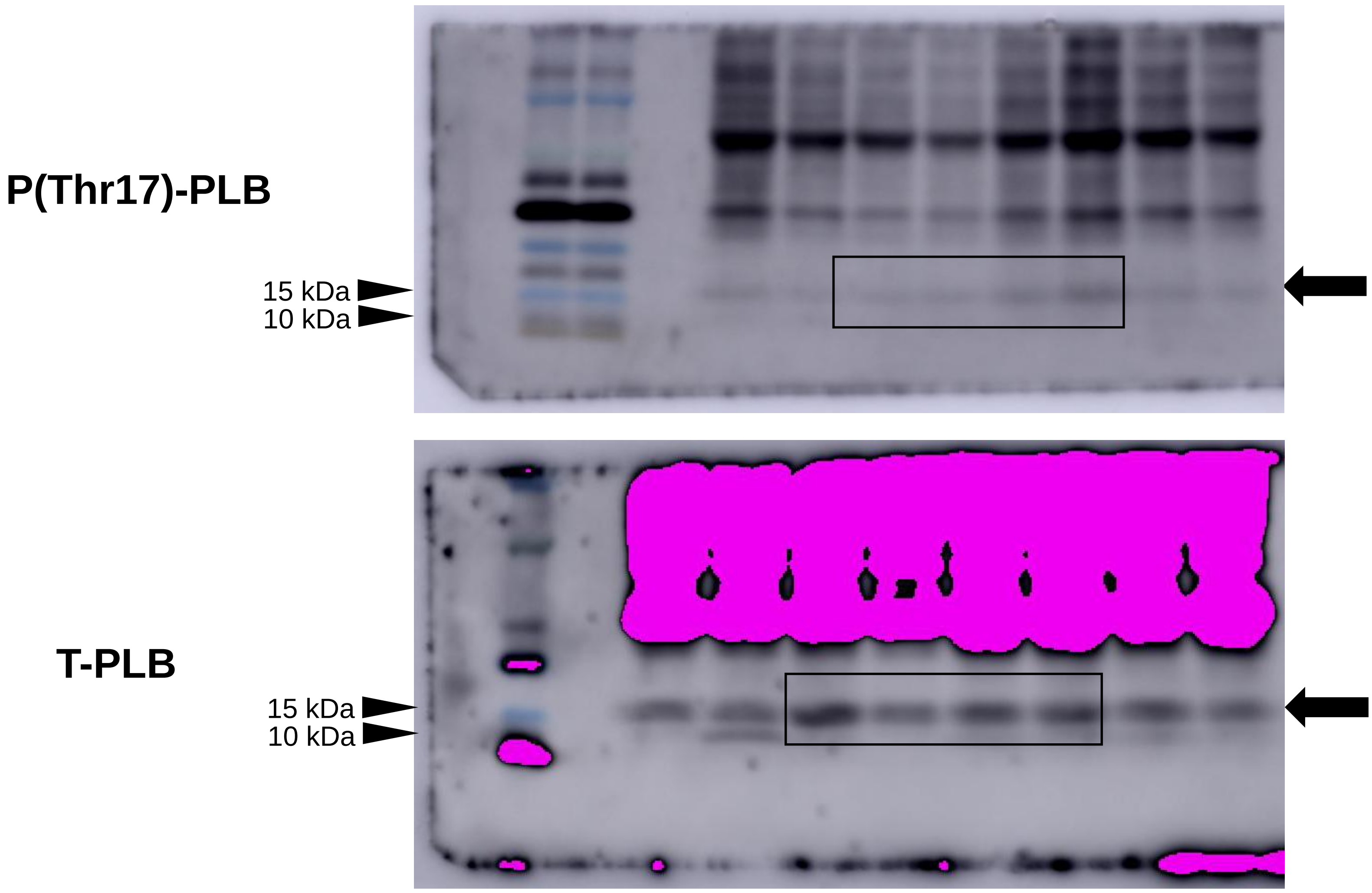

Uncropped western blots used for figure 7 (a)

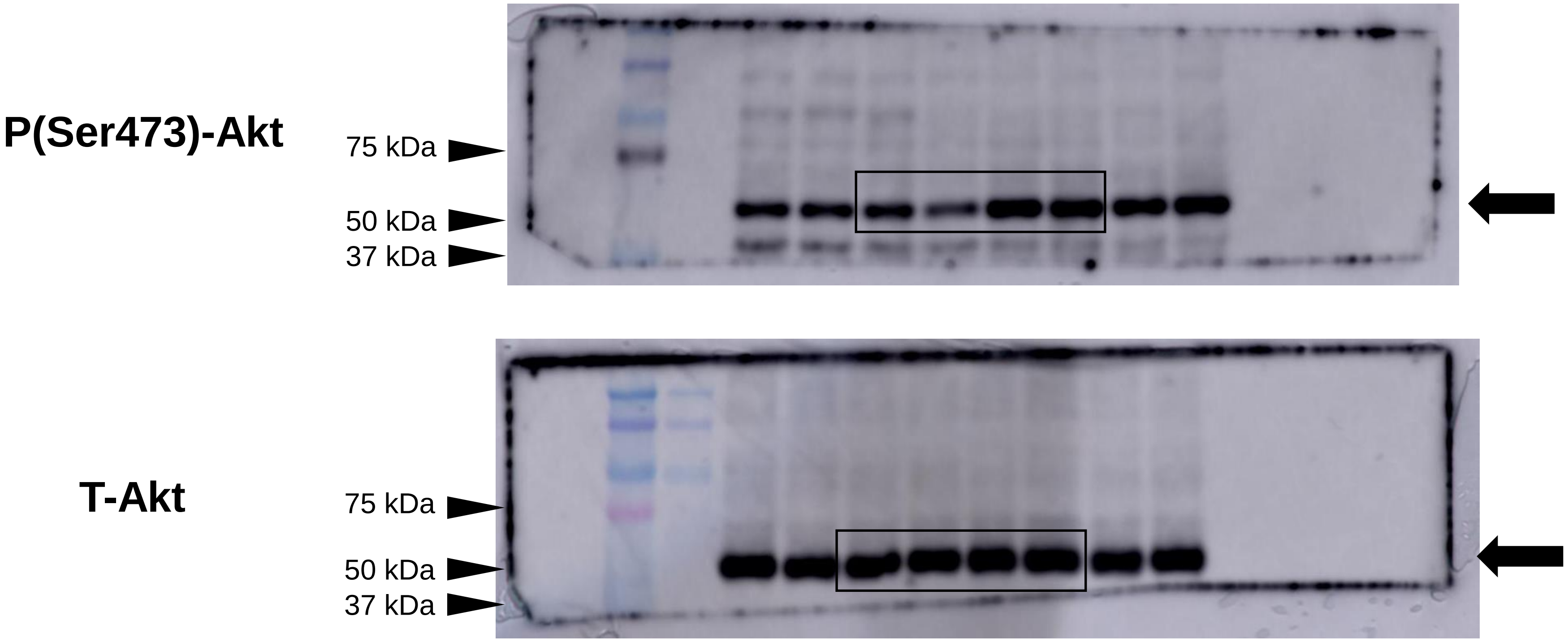

Uncropped western blots used for figure 7 (b)

P(Ser2448)-mTOR

T-mTOR

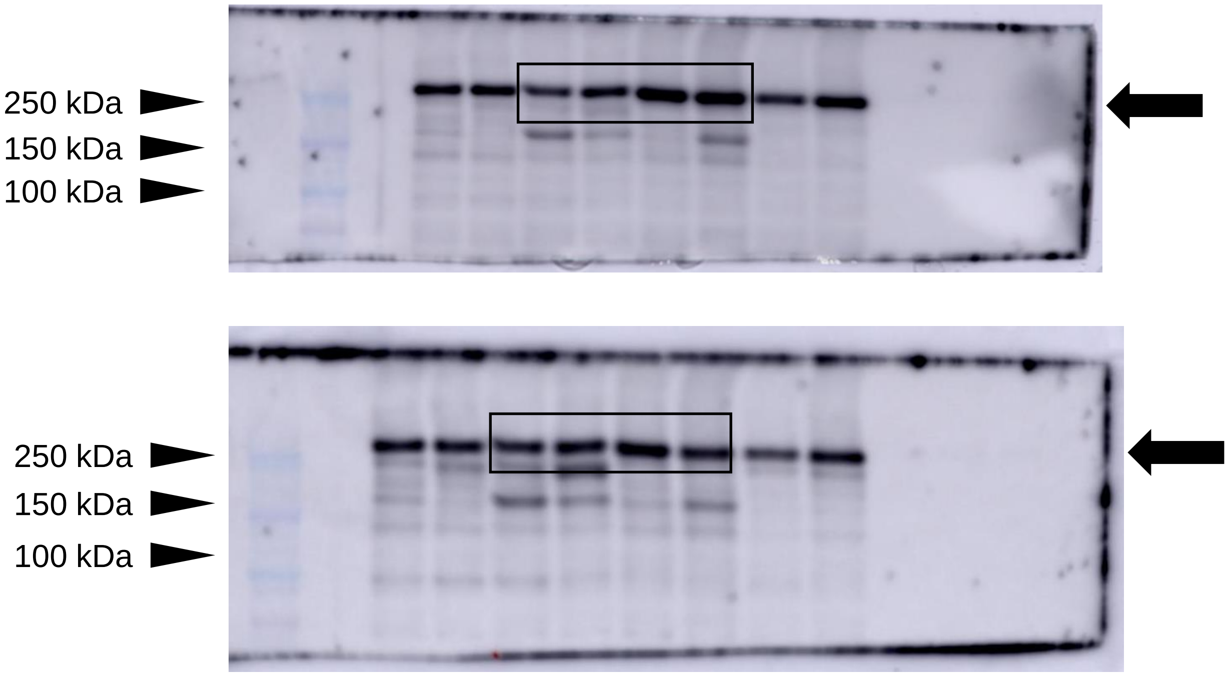

Uncropped western blots used for figure 7 (c)

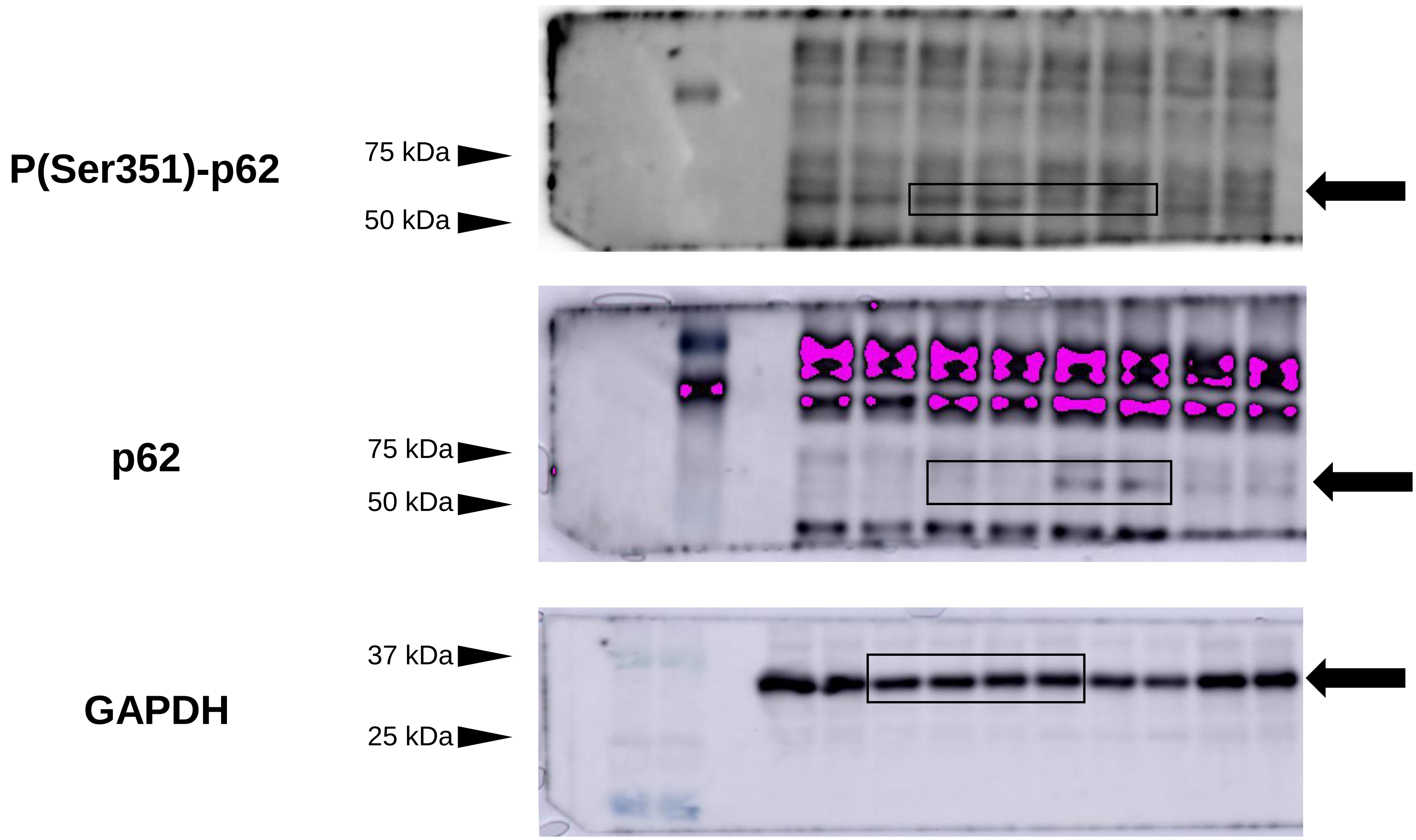

Uncropped western blots used for figure 7 (d)

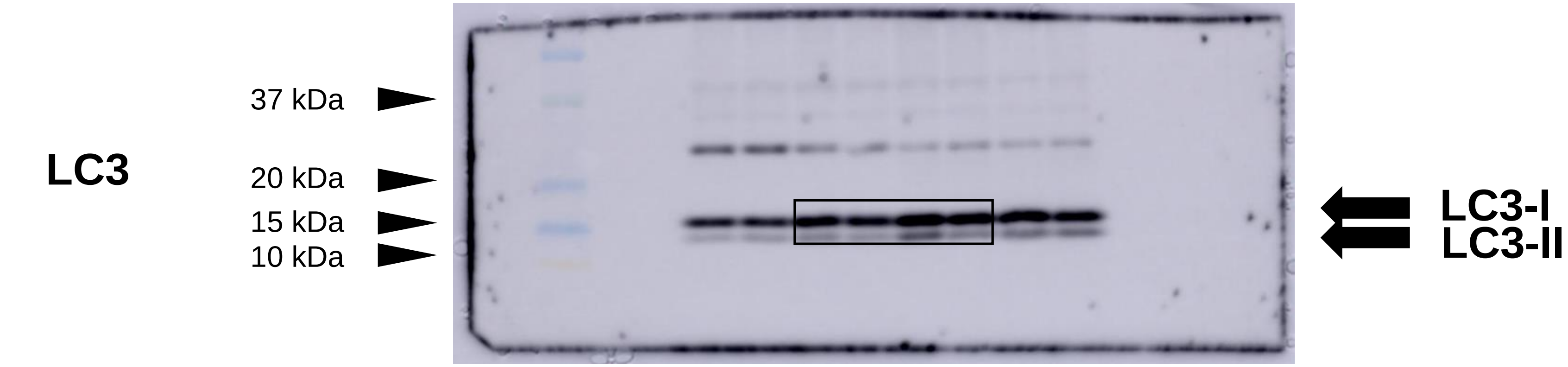

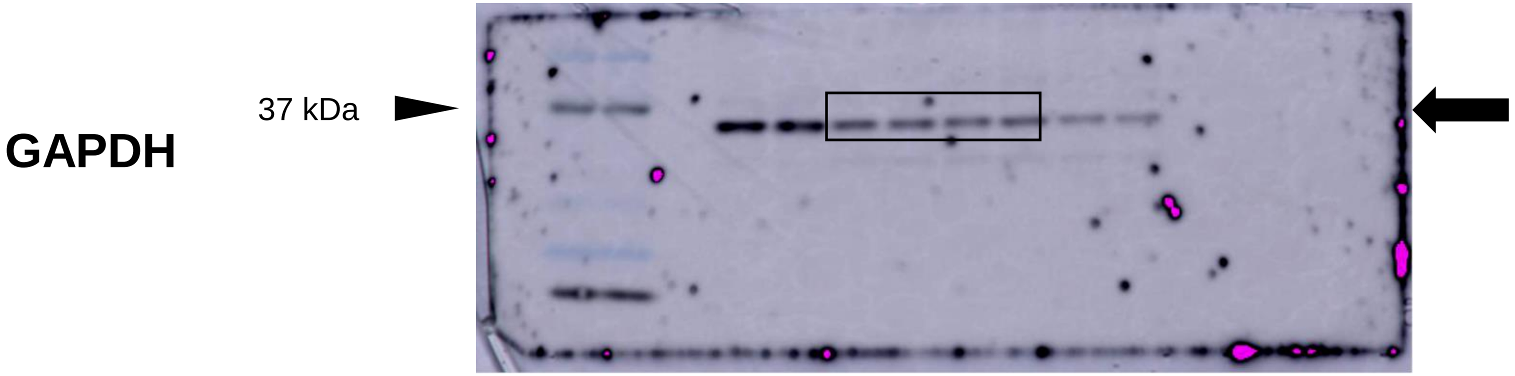

Supplement: Supplementary file 2 — Data S2. [file PHY2-13-e70677-s002.pdf]
